# Optimized measurements of separations and angles between intra-molecular fluorescent markers

**DOI:** 10.1038/ncomms9621

**Published:** 2015-10-16

**Authors:** Kim I. Mortensen, Jongmin Sung, Henrik Flyvbjerg, James A. Spudich

**Affiliations:** 1 Department of Biochemistry, Stanford University School of Medicine, Stanford, California 94305, USA; 2 Department of Micro- and Nanotechnology, Technical University of Denmark, Kongens Lyngby DK-2800, Denmark; 3 Department of Applied Physics, Stanford University, Stanford, California 94305, USA

## Abstract

We demonstrate a novel, yet simple tool for the study of structure and function of biomolecules by extending two-colour co-localization microscopy to fluorescent molecules with fixed orientations and in intra-molecular proximity. From each colour-separated microscope image in a time-lapse movie and using only simple means, we simultaneously determine both the relative (*x*,*y*)-separation of the fluorophores and their individual orientations in space with accuracy and precision. The positions and orientations of two domains of the same molecule are thus time-resolved. Using short double-stranded DNA molecules internally labelled with two fixed fluorophores, we demonstrate the accuracy and precision of our method using the known structure of double-stranded DNA as a benchmark, resolve 10-base-pair differences in fluorophore separations, and determine the unique 3D orientation of each DNA molecule, thereby establishing short, double-labelled DNA molecules as probes of 3D orientation of anything to which one can attach them firmly.

## Abstract (web summary)

Single molecule super-resolution microscopy can reliably localize objects with high precision, but the accuracy can be called into question. Here the authors present a methodology to accurately and precisely measure nanometre separation and orientation of two fixed fluorophores from colour-separated images.

## Graphical abstract

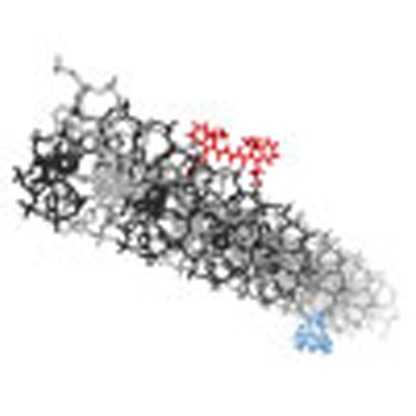

In localization-based single-molecule studies and super-resolution microscopy, fluorophores are imaged in a microscope. Owing to fundamental diffraction effects, individual fluorophores image as diffraction-limited spots. To directly resolve two such spots in one image, they must be separated by at least their half-width, typically ∼200 nm (Abbe's law). This limit on resolution can be circumvented by separating imaged spots either in time, by switching the fluorophores between active and inactive fluorescent states (STORM, PALM)^1^ or in frequency space, by using fluorophores of different colours as in single-molecule high-resolution co-localization^2^. Either way, the isolated spot has an intensity distribution that is a realization of the fluorophore's theoretical point spread function (PSF). Consequently, one may fit the latter to the measured intensity distribution and thus localize the fluorophore with a precision that increases with the number of photons in the imaged spot. With this method, fluorescent probes are routinely localized with nanometre precision^1^,^2^,^3^,^4^,^5^,^6^, that is, nanometre reproducibility.

For such precision to be useful, accuracy is critical. Accuracy quantifies systematic errors, that is, agreement of the localized position with its true value in the absence of stochastic errors^6^. It is very sensitive to proper choice of theoretical PSF, and the latter depends on experimental conditions. Thus, if a fluorophore is bound to a molecule of interest with a single, flexible linker and can change its orientation unhindered by its environment, its rapid thermal motion will cause it to image as an isotropic superposition of itself. Consequently, its theoretical PSF is very well approximated with a two-dimensional (2D) Gaussian plus a constant ‘background'^4^. When this approximation is justified, localization is accurate^2^,^5^, and even sub-nanometre accuracy has been demonstrated with advanced feedback-controlled instruments^5^.

A fluorophore bound with a single, flexible linker may be constrained in its thermal motion, however, because of a crowded molecular environment^7^,^8^, for example, or interactions with neighbouring proteins or nucleotide residues through hydrophobic or stacking effects^5^,^9^. Or the orientation of a fluorophore may be fixed deliberately to a molecule of interest^4^,^10^,^11^,^12^,^13^. In both cases, the PSF is typically anisotropic, and if it is approximated by a 2D Gaussian, localization accuracy is compromised: Systematic errors may amount to tens of nanometres^14^,^15^, irrespective of precision, which may still be nanometres. Thus, the true position of the fluorophore can be tens of nanometres away from the precisely estimated position.

Such anisotropic intensity distributions provide information about the fluorophore's orientation, however. Fluorophores with fixed orientation have been analysed to extract fluorophore orientations^4^,^7^,^8^,^10^,^11^,^12^,^13^,^16^,^17^,^18^,^19^, for example, using deliberately defocused images^11^,^12^,^13^, polarization-based measurements^10^ or engineered PSFs^17^. In some cases, the positions were also extracted^4^,^11^,^12^,^13^,^17^,^18^,^19^. Unfortunately, in some instances^11^,^12^, positions were inaccurate because a 2D Gaussian was fitted to focused images of fixed fluorophores to extract locations^14^,^15^. Importantly, neither of these reports of methods developed in other laboratories^7^,^8^,^10^,^11^,^12^,^13^,^16^,^17^,^18^,^19^ nor our previous work^4^, which demonstrated optimal precision, provided direct, rigorous experimental verification of accuracy (with precision) for estimates of orientation and location of probes, for example, in the form of an experimental demonstration that estimates of positions and orientations of fluorophores agree with the experimental reality.

However, if optimally analysed, these anisotropic intensity distributions may provide positions and orientations of fluorescent probes with nanometre, respectively, degree, precision and accuracy. We demonstrate this below, and in that process we extend current experimental practice with fixed fluorophores to a two-colour assay^2^. This allows us to determine separation and orientations simultaneously for two fluorophores in intra-molecular proximity from a single spectrally separated image of them. Based on that we verify experimentally that the methodology is (i) accurate, by demonstrating that its estimates of distances and relative orientations between two fixed fluorophores agree with the experimental reality, and (ii) about as precise as the information limit allows^4^. This establishes the simultaneous use of two fixed fluorophores of different colour as a novel structural tool for the study of molecular conformation by allowing measurements of orientation and position of one molecular domain with respect to another.

## Results

### Analysis of images of single fixed fluorophores

A fluorophore emits photons via a dipole transition, and if the dipole is fixed in space, the diffraction-limited image of the fluorophore (Supplementary Fig. 1) depends radically on the polar and azimuthal angles of that emission dipole's orientation in space and on the objective's distance from focus^4^,^11^,^12^,^13^,^20^. Extending our previous work^4^, we here use the theoretical PSF with maximum likelihood estimation (MLE) to extract simultaneously the position and the orientation of a molecule, directly from its nearly focused image (Methods). In that process, we also determine the total number of source photons emitted by the probe, a constant background level, and now additionally the objective's distance from its focus (Methods, Supplementary Fig. 1). Although the latter does not directly carry information about the molecule, its determination is paramount for accurate estimates of the molecule's position and orientation, we show. This general procedure of MLE based on a theoretical PSF was dubbed MLEwT (Maximum Likelihood Estimation with the Theoretical PSF) in ref. 4. Thus, we could refer to the present method as ‘MLEwT applied to fixed fluorophores and with the focal depth included as a fitting parameter.' For brevity we instead will refer to it as diPOLE (dipole-based Parametric Orientation and Location Estimation).

### Two-colour localization microscopy with fixed fluorophores

**Figure 1:**
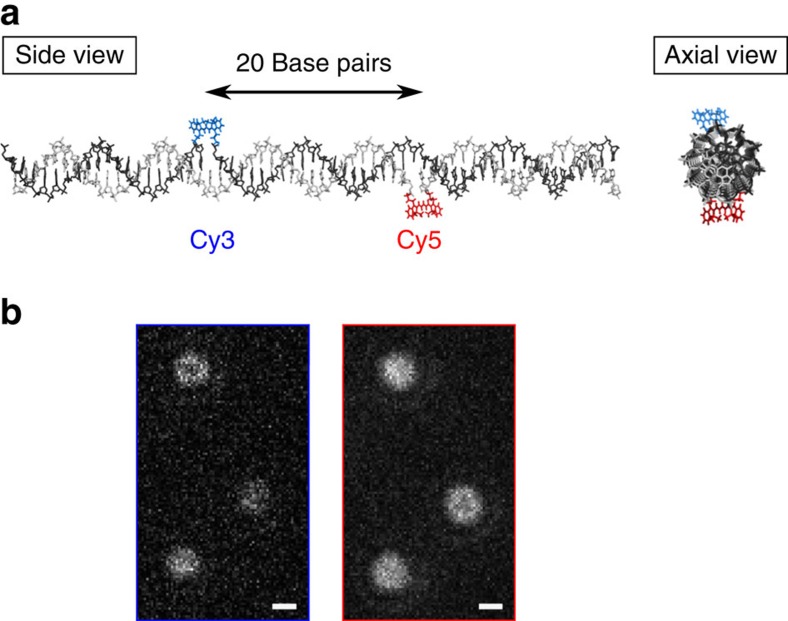
Double-stranded DNA (dsDNA) labelled internally with two fluorophores of different colour. (**a**) Schematic illustration of the B-form dsDNA sample with internally labelled Cy3 and Cy5 dyes covalently attached to the backbone. The total length of each strand is 60 bp, and Cy3 and Cy5 labels complementary strands 20 and 21 bp from each 5′ end, respectively, which makes the inter-dye separation 20 bp. We also tested our method on a similar construct with a total length of 90 bp and a 30-bp inter-dye separation. (**b**) Anisotropic diffraction-limited spots from Cy3 and Cy5 internally labelling dsDNA molecules at 20 bp separation as in **a**. The light from each fluorophore pair (here three pairs) was spectrally separated and imaged on two different areas (Cy3, left; Cy5, right) of an EMCCD camera (Methods and Supplementary Fig. 2). Scale bars, 300 nm.

For demonstration of the theoretical analysis and to experimentally establish two-colour co-localization microscopy with fixed fluorophores as a structural tool, we used two constructs of double-stranded DNA (dsDNA), in which Cy3 and Cy5 are covalently fused into the backbone of each complementary strand with double attachments (Fig. 1a, Methods). The orientation of each fluorophore is fairly rigidly fixed relatively to the dsDNA backbone^21^. The distance and relative orientation between the two dye molecules in a construct are controlled by the number of base pairs between them; we used 20 and 30 bp separations. The dsDNA was non-specifically immobilized on a poly-L-lysine (PLL)-coated microscope coverslip, and the fluorescent probes were imaged using a conventional total internal reflection fluorescence (TIRF) microscope, with some modifications (Methods and Supplementary Fig. 2). The probes are separated ∼7 nm, respectively, ∼10 nm, which is just a few per cent of the diffraction limit, and even within the range of single-molecule fluorescence resonance energy transfer^22^. Therefore, we spectrally separated the light emitted from Cy3 and Cy5 and imaged them on two different areas (channels 1 and 2, respectively) of an EMCCD camera (Fig. 1b, Methods and Supplementary Fig. 2).

**Figure 2:**
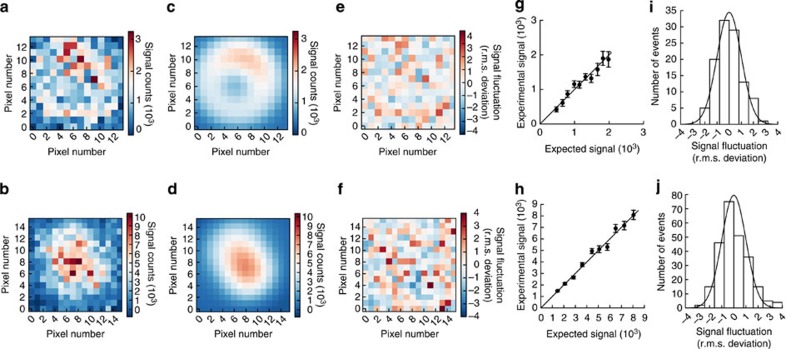
Point spread functions for Cy3 and Cy5 fluorophores internally labelling complementary strands on the same dsDNA molecule (Fig. 1) at 20 bp separation. ‘Signal counts' denotes the raw output signal from the EMCCD camera's pixels, not to be confused with photon counts (Methods). (**a**,**b**) TIRF microscopy images of Cy3 and Cy5 fluorophores, respectively (Methods). (**c**,**d**) Theoretical images with parameter values obtained by applying diPOLE to fixed probe images in **a** and **b**, respectively, and using the dyes' average emission wavelengths of 580 and 670 nm. We found orientations in polar angles for **c** and **d** of 25° and 54°, respectively, and in azimuthal angles of 69° and 299°, source photon numbers of ∼3,200 and ∼9,500, and values of the defocus of 46 and −74 nm. (**e**,**f**) Rescaled residuals. Each pixel shows the difference between measured and expected pixel signal values in units of its theoretical root mean squared (r.m.s.) deviation as given by the colour bar. (**g**,**h**) Measured signal values compared with expected values. We binned the expected signals and associated pixels with a bin if the expected signal fell in it. For each bin, the mean experimental signal is plotted against the expected signal with an error bar indicating the theoretical s.e.m. Data points shown should scatter about the straight line through the origin with unit slope, each in a Gaussian manner with s.d. equal to the shown error bar. (**i**,**j**) Histograms of rescaled residuals. For sufficiently large expected pixel signals, theory predicts the standard normal distribution (solid line) for the fluctuations in **e** and **f**, respectively.

Each channel's image (Fig. 2a,b) was fitted individually using diPOLE (Methods, Supplementary Software). This yielded the fluorophores' locations in the sample plane and their spatial orientations. The experimental intensity distributions (Fig. 2a,b) and the corresponding theoretical distributions (Fig. 2c,d) are in near-perfect agreement (Fig. 2e–j), showing that the theory assuming full fluorophore fixation is appropriate for the present experimental practice.

### Calibration of the map between colour-separated images

Identical locations in the two channels were mapped to each other using fiducial markers: multi-coloured fluorescent beads that emit into both channels. We non-specifically attached such beads to the PLL-coated coverslip surface at random positions. Their approximate positions in both channels were determined automatically and then localized with nanometre precision using the Gaussian Mask Estimator (GME^4^,^23^; Methods and Supplementary Fig. 3). The difference between a bead's simultaneous coordinates in the two images was unaffected by sample drift, and only fluctuated through a movie because of finite photon statistics (shot noise). We used this statistics for quality control, to eliminate less firmly attached beads: we conservatively selected beads with separations that were in agreement with a normal distribution around their mean separation with s.d. given theoretically by the number of photons in its images^4^ (Supplementary Fig. 4). This qualified 5,622 out of 7,324 imaged beads as fiducial markers for the map between the two channels. The difference between a bead's coordinates in the two channels was negligibly auto-correlated in time, as expected when photon shot-noise is responsible for fluctuations (Supplementary Fig. 5). Thus, the time-averaged separation has s.e.m.=s.d./✓*N*, where *N*=90 is the number of frames in a movie. This reduced shot noise errors on fiducial markers to negligible sub-nanometre levels (Supplementary Fig. 5).

The resulting map of fiducials between channels reveals smooth spatial variations across the imaging region, but superposed with a speckled pattern of variations with characteristic length-scale near the diffraction limit, presumably due to imperfections or aberrations in the optical system on a shorter length-scale (Supplementary Figs 6 and 7). To minimize speckle effects in our map between channels, we mapped each coordinate with a locally determined second-degree polynomial based on an excess of fiducial beads in a region around a position of interest (Methods). An excess of fiducial beads was necessary, because the physical centres of the two relevant dyes are offset relatively to each other in the individual bead (Supplementary Fig. 8). By using an excess of beads in a map, their randomly oriented offsets of colour centres cause errors with random signs, that is, they almost cancel each other (Methods and Supplementary Fig. 8). The resulting map between channels had ∼2 nm errors (Supplementary Fig. 8).

Another concern is the possibility of slow drift of the optics in the emission paths^5^ (Supplementary Fig. 2). This could be detrimental to the accuracy of the map between channels. To detect and correct for this, we first measured the imaging positions of the emission paths by recording bright light passing through the imaging windows of the two channels (Supplementary Fig. 2). This amplified the contrast to the background at the windows' edges (Supplementary Fig. 9). We measured the positions of these edges, by fitting a theoretical light intensity distribution for diffraction by a rectangular window to the measured distributions (Methods and Supplementary Fig. 9). This procedure determined edges and allowed correction for drift in the emission path, both with nanometre precision (Methods and Supplementary Fig. 9).

### Measurements of intra-molecular distances and orientations

**Figure 3:**
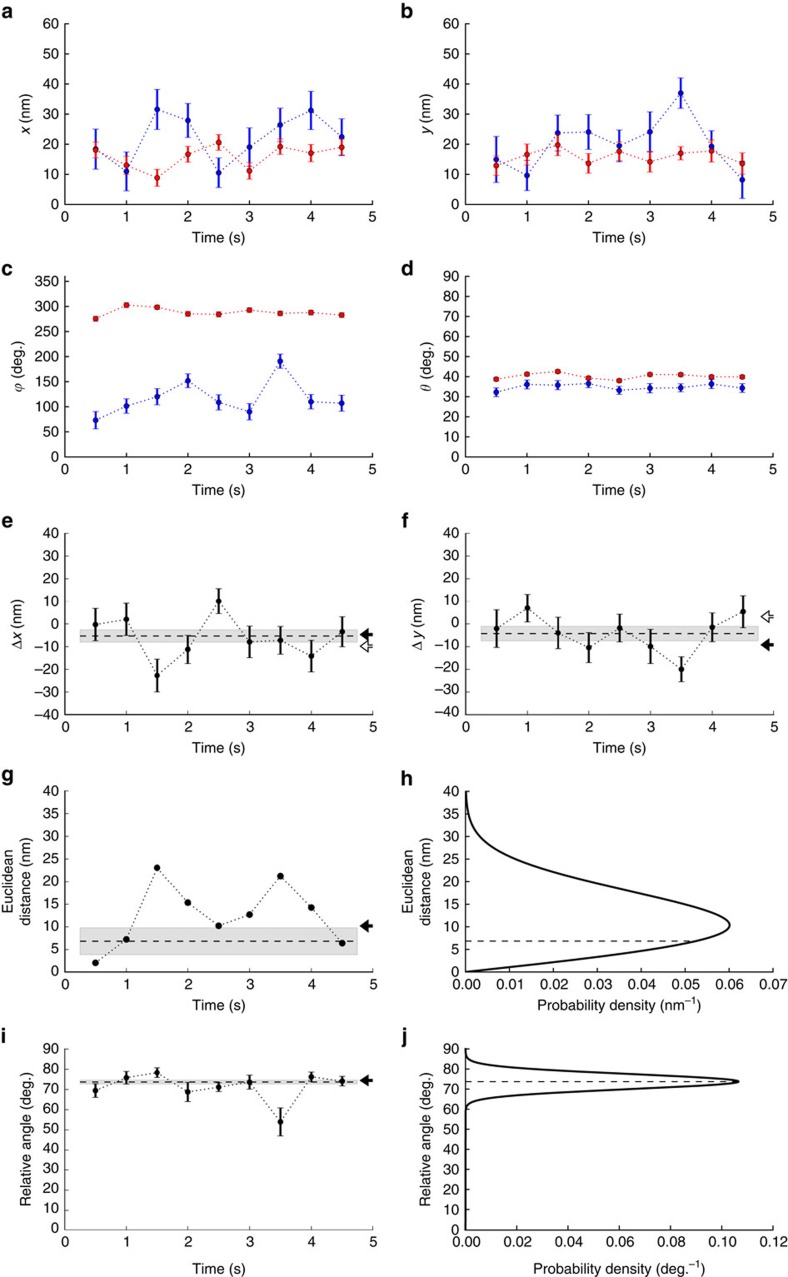
Distance and angle between a pair of fluorophores fixed in one DNA molecule, estimated using diPOLE. (**a**) Time series at 2 Hz of repeatedly estimated position coordinates (*x*) of a pair of fixed Cy3 (blue circles) and Cy5 (red circles) fluorophores, attached internally to the same dsDNA molecule at 30 bp separation (Fig. 1a). For comparison, coordinates were mapped between image channels as described in the text. Error bars assume theoretical shot noise as only source of error. (**b**) Same as **a** for position coordinate *y*. (**c**) Same as **a** for the azimuthal angle (*ϕ*). (**d**) Same as **a** for the polar angle (*θ*). (**e**) Difference in *x*-coordinates (black circles) from **a**. We calculated the weighted mean value (dashed line) and the theoretical s.e.m. (grey region). The data are consistent with a normal distribution centred on this mean value. The arrows indicate two predictions for the *x*-coordinate difference based alone on the estimated angles in **c**,**d** and the dsDNA structure (Supplementary Fig. 13). (**f**) Same as **e** for the difference in *y*-coordinates. (**g**) Euclidean distance between the fluorophores, estimated frame-by-frame (black circles). The black arrow indicates the expected value from the dsDNA structure. We estimated the Euclidean distance between the probes (dashed line) as the Euclidean length of the movie-average of the frame-by-frame vector difference between the probes shown in **e**,**f**. Grey region shows the theoretical s.d. of this estimate. (**h**) Non-Gaussian distribution^24^ (solid line) of frame-by-frame Euclidean distances, resulting from probe separation (dashed line) being comparable to statistical errors on its estimates in **g**. Note that this distribution peaks well above its true distance. (**i**) Relative angle between the emission dipoles of the probes. Its expected value based on the dsDNA structure is indicated (black arrow). We calculated the weighted mean value (dashed line) with theoretical s.e.m. (grey region). (**j**) Distribution of relative angles (solid line). In general, it is a non-Gaussian distribution (Supplementary Note 1), but here it is indistinguishable from a Gaussian centred on the estimate from **i**. Consequently, the relative angle is estimated as the movie average over estimates in individual frames shown in **i**. deg., degree.

We selected co-localized pairs of Cy3 and Cy5 dyes, assuming they were from the same dsDNA molecule, as was likely from the relatively sparse distribution of dsDNA molecules on the surface (Fig. 1b, Methods). From each frame in a time-lapse movie of the probes, we simultaneously estimated each probe's position coordinates in the *xy*-plane (Fig. 3a,b) and angles of dipole orientation (Fig. 3c,d). From these we calculated the probe separation in the *xy*-plane (Fig. 3e–h) and the relative angle between them (Fig. 3i,j). These measures allow us to compare intra-molecular distances and orientations among the randomly oriented dsDNA molecules. The vector difference between the position coordinates of the two probes in a pair (Fig. 3e,f) was unaffected by sample drift during a movie. Each vector difference scattered normally around its mean values with an s.d. that agreed with the minimal value allowed by the information limit given theoretically by the number of photons in probe images (Methods and Supplementary Figs 10 and 11). We calculated the 2D Euclidean distance between probes in a pair from their time-averaged vector differences (Fig. 3g). It agrees with the value expected from a simple model of the dsDNA structure (Fig. 3g and Supplementary Fig. 12). It is important to average vector differences before calculating the Euclidean distance. If the Euclidean distance between probes in a pair is calculated frame-by-frame in a movie and then averaged, this average overestimates the true Euclidean distance^24^ (Fig. 3h). Also, had the analysis falsely assumed that the dyes were imaged exactly in focus, the resulting distance estimate would have been significantly compromised: in that case, each coordinate of an estimated position would be subject to a systematic error that depends on the focus mismatch and the polar angle of the probe. Such systematic errors may exceed 10 nm for a position coordinate per probe for intermediate polar angles and typical values of the objective's distance from the focus (Methods and Supplementary Fig. 10).

Fluorophores were approximately fixed in orientation during imaging, as demonstrated by the reproducibility of the estimated azimuthal and polar angles of the probes (Fig. 3c,d). (We discarded fluorophores that were not.) From these angles, we calculated the relative angle between probes in a pair (Fig. 3i), which is independent of all drift and of pitch, yaw and roll of the dsDNA molecule. The relative angles scatter tightly around their mean value (Fig. 3i), but typically do so with an s.d. that is slightly larger than the one expected from photon shot noise alone (Supplementary Figs 10 and 11). This slack compromised neither the accuracy of the estimate nor the Gaussian approximation of the distribution of the relative angles (Fig. 3j and Supplementary Note 1). Justified by the latter, we calculated the mean relative angle between probes and its s.e.m. from the Gaussian distributed relative angles. In the single molecule analysed in Fig. 3 with probes separated by 30 bp as an example, diPOLE applied to the fluorescent probes yields a Euclidean distance and relative angle between them that is in fine agreement with a simple model of the dsDNA structure (Fig. 3 and Supplementary Fig. 12). The agreement is sufficiently good to allow determination of three-dimensional (3D) orientation of the dsDNA molecule: the estimated fluorophore orientations alone specify two possible 3D orientations of the assumed model dsDNA structure (Supplementary Fig. 13), where the degeneracy is due to the fact that the emission dipoles are not directional. However, each of the two orientations predicts a vector difference for the fluorophore positions, and those we have measured (Fig. 3e,f). Comparison of the measured vector differences to the predicted values from the two candidate orientations singles out one orientation (black arrows, Fig. 3e,f) with odds ∼20 (Methods and Supplementary Fig. 14) over the other (white arrows, Fig. 3e,f). Thus, diPOLE extracts the full 3D orientation, that is, pitch, yaw and roll, of the dsDNA molecule.

**Figure 4:**
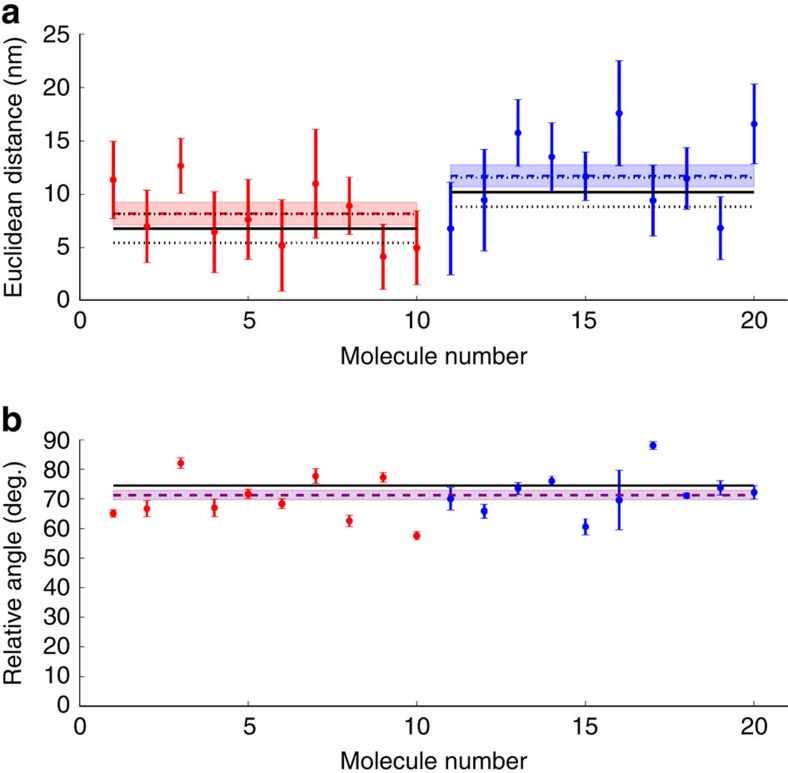
Demonstration of the accuracy and precision of diPOLE. (**a**) Euclidean distance estimates between probes at 20 bp (red circles) and 30 bp (blue circles) separation, respectively. Error bars are theoretical, assuming shot noise as only source of error. The expected Euclidean separation based on the dsDNA structure (black solid lines) is 6.8 nm and 10.2 nm, for 20 and 30 bp separation, respectively, with an uncertainty (dotted lines) because of minor and major groove interactions between fluorophores and dsDNA (Supplementary Fig. 12). We find weighted mean separations of, respectively, 8±1 and 12±1 nm (mean±theoretical s.e.m.; dashed lines with coloured area), which are in perfect agreement with the expected values (black solid lines) and in tantalizing agreement with the values expected if both probes are stuck in their extremal positions in the minor groove (dotted lines coinciding with dashed lines). (**b**) Same as **a** for relative angles. Error bars are experimentally calculated s.e.m. The expected relative angle between the probes (black solid line) is 74° in both samples because they differ in probe separation by a full repeat of the dsDNA. There is no discernable difference between the relative angles measured in the two samples. We found a mean orientation of 71±2° (mean±experimental s.e.m.; purple dashed line with purple area), which agrees with the expected value. The individual angle estimates scatter more than photon shot noise alone predicts. We therefore report here the experimental s.e.m. for realism. deg., degree.

Distances and relative angles were measured over time for 10 molecules with 20 bp separations and 10 molecules with 30 bp separations (Fig. 4a,b). We calculated the average separation for each sample and found 8±1 nm (mean±theoretical s.e.m.) for probes separated by 20 bp and 12±1 nm (mean±theoretical s.e.m.) for probes separated by 30 bp. The distance estimates for each group of molecules scatter about their respective mean separations (Fig. 4a) with an s.d. that is consistent with the information limit. The population means agree with the values expected from a simple model of the internally labelled dsDNA (Supplementary Fig. 12), that is, with expected separations of 6.8 nm (20 bp) and 10.2 nm (30 bp). The latter values are subject to small uncertainties because of possible interactions of the dyes with the major or minor grooves of the dsDNA (Fig. 4a and Supplementary Fig. 12). This demonstrates that diPOLE applied to a single pair of probes provides an accurate and precise estimate of their separation at the nanometre level and that diPOLE applied to a small number of probe pairs discriminates a 10 bp difference.

The expected relative angle between the probes is identical in our two samples (Fig. 4b and Supplementary Fig. 12), because they differ in probe separation by a single repeating unit of the dsDNA helix. Each molecule yields a mean relative angle, which is determined so precisely (Figs 3i and 4b) that discernable systematic variation between molecules is revealed (Fig. 4b). This is likely due to dye–surface and/or dye–DNA interactions. An interaction resulting in an angstrom change of one end of a fluorophore may give rise to a ∼5° change in relative orientation. This easily explains the excess scatter of the mean relative orientations in Fig. 4b. For the population, we found a mean value of 71±2° (mean±experimental s.e.m.), where this s.e.m. predominantly reflects the systematic variation in the results likely due to surface interaction. This result agrees with the expected relative angle of 74° based on the simple dsDNA model structure. Finally, 17 of the 20 molecules were sufficiently unaffected by surface interaction to comply well with the assumed model dsDNA structure, quantified by the agreement of measured vector differences with their predictions from the fluorophore orientations alone (Supplementary Fig. 14). We determined the 3D orientation for each of those (Supplementary Fig. 14).

## Discussion

We have previously shown that a theoretical PSF in conjunction with MLE provides an optimally precise method for estimating the position and orientation of a fixed fluorescent probe^4^. Here we have critically extended that method and we have demonstrated that the resulting method, diPOLE, is precise and accurate to within a few nanometres and a few degrees, when applied to fixed fluorescent probes attached to a single molecule.

The scatter of estimates of the mean probe distance obtained with diPOLE (Fig. 4a) is fully explained by photon shot noise and the finite number of beads included in the calibration of the map between the channels. Thus, distances measured with diPOLE are optimally precise. Specifically, we found precisions of mean distance estimates for individual molecules of ∼2–5 nm. Using additional, or brighter, images of a fluorophore pair would increase this precision. Doing that would also reduce the standard error of the population-averaged distances below their current values of ∼1 nm. So would inclusion of additional molecules in the analysis. Similarly, the ∼2 nm precision of each map between channels would improve by inclusion of additional beads in map calibrations. In each of these cases, precision would improve as one over the square root of the relevant statistical quantity, that is, the number of photons, images, molecules or beads. The current resolution, however, is sufficient to establish both nanometre accuracy and precision of distances measured within an individual molecule with diPOLE. When such distances are averaged over a small number of molecules, a 10-bp difference, between 20 and 30 bp, is resolved, we have shown.

On the other hand, diPOLE yields mean estimates of the relative angle between the probes (Fig. 4b) that are so precise that a molecule-to-molecule variation is revealed, which, however, is on the order of a few degrees and easily explained by, for example, surface interaction. For improvements of these results, likely a better benchmark than our dsDNA construct is required, not improved statistics.

At 2 Hz, we recorded ∼1,000–20,000 photons per image, depending on the fluorophore's orientation with respect to the excitation light field, its position in the field of view and its possible function as a fluorescence resonance energy transfer donor or acceptor. This suggests that the method will have a time resolution that is an order of magnitude better using similar excitation laser intensity, if one considers only the brightest probes. Increased excitation laser power will also improve time resolution, albeit at the expense of an increased rate of photo bleaching.

All this makes fixed fluorescent probes a reliable tool in single- and dual-colour assays for simultaneous probing of positions and orientations directly from images. With a single-colour assay, macromolecular dynamics that involves translational and/or rotational motion can be monitored. This could, for example, be used to improve the resolution of measurements of lever-arm dynamics of processive molecular motors, such as myosin V and myosin VI (refs 10, 11, 12). A two-colour assay enables dynamic studies of changes in relative orientation and position, for example, originating from intra- or inter-macro-molecular interactions, between discrete molecular states that are sufficiently rigid to ensure fixation of the spatial orientation of the fluorescent probes. Also the probe-dsDNA construct itself (Fig. 1a) may find use as an advanced dual-colour fluorescent probe to be attached to a target molecule, for example, a DNA-binding protein, in cases where the full 3D orientation of the latter is of interest. Furthermore, following our example, that construct provides a robust structure for future benchmarking of other methods for extracting orientation and location from fixed fluorophores. All that is now possible with ease and confidence and a resolution that was inaccessible till now in single-molecule studies.

## Methods

### dsDNA samples internally labelled with Cy3/Cy5 dyes

We purchased dsDNA samples with internally labelled Cy3 and Cy5 dyes with 20 and 30 bp separations from Integrated DNA Technologies, which were all synthesized, hybridized and HPLC purified before use. The total numbers of base pairs are 60 and 90 bp, for the 20 and 30 bp samples, respectively. Cy3 and Cy5 were labelled in the middle of each complementary strand such that the two dyes are separated by either 20 or 30 bp along the axial direction (Fig. 1a). The sequences for the constructs were randomly generated with the ratio of AT/GC=2:3, but a couple of base pairs near the Cy3 and Cy5 dye locations and at the ends of the dsDNA were replaced for more GC pairs to stabilize of the fixation of the dyes. The dyes are embedded in the double-helix structure without a base-paring partner, so stable base-paring of the neighbouring nucleotides are important for their stable fixation (Supplementary Table 1).

### Fluorescent beads for two-channel mapping calibration

For the mapping calibration between the two channels, we used TetraSpeck fluorescent beads with a diameter of 100 nm (Invitrogen, T7279), which are coated with four different dyes. The density of the original stock was ∼1.8 × 1,014 particles *L*^−1^=300 pM. It was diluted in buffer (1:30 for 10 pM final concentration), vortexed and sonicated for 1 min before use. Here, only the orange and dark red dyes were used, with excitation by two lasers, 561 and 643 nm. Diffraction-limited spots from the beads were imaged with two different channels in the imaging system (Supplementary Figs 2 and 3).

### Buffers

All buffers contained 4 mM Tris-HCl (pH 8) and 1 mM MgCl_2_. Presence of the divalent salt increased stability of the dsDNA attached to the PLL-coated coverslip surface. We used an imaging buffer, containing 50 nM protocatechuate-3,4-dioxygenase (Sigma-Aldrich, P8279), 25 mM protocatechuic acid (Sigma-Aldrich, 37580), 0.4 % glucose, 0.1 mg ml^-1^ glucose oxidase (Calbiochem, 345386), 0.02 mg ml^−1^ catalase (Calbiochem, 219261) and 2 mM Trolox (Sigma Aldrich, 23881-3), for imaging the Cy3/Cy5 single molecules with enhanced photo-stability and brightness. Machine-filtered pure water (EMD Millipore, Milli-Q) was used throughout.

### PLL-coated coverslips

Glass coverslips (Fisher Scientific, #1, 22 × 22 mm^2^) were rinsed with machine-filtered water, dried with filtered nitrogen gas, and incubated in a ultraviolet ozone cleaner (Jelight, UVO Cleaner 42) for 30 min. The cleaned coverslips were incubated in 0.01 % PLL solution (EMS, 19320-A) for 5 min and dried overnight on coverslip staining outfits (Thomas Scientific, 8542E40). The negatively charged dsDNA constructs were non-specifically and electrostatically attached to the PLL-coated coverslip surface. The condition of the dried PLL coverslip usually remained good for a few weeks, but the data in this paper were collected with freshly prepared coverslips.

### Microscope

The experiment was conducted on a conventional inverted TIRF fluorescence microscope (Nikon, Eclipse TE2000-PSF) with an oil immersion TIRF objective lens (Nikon, Apo-TIRF 100x, NA 1.49; Supplementary Fig. 2). We used the built-in Perfect Focus System (PFS) to minimize possible focus drift and/or fluctuations during the measurement. We adjusted the focal plane by using the offset lens in the PFS such that the diffraction-limited spots from the TetraSpeck beads or single molecules were tightly focused with minimum width. The microscope sample slide was firmly mounted on a sample holder on a *XYZ*-motorized stage with a Z-axis piezo stage (ASI, PZ-2000). The *XYZ*-motorized stage was mainly used to move the sample in *XY* plane and to roughly adjust the focus in *Z*-axis, whereas the *Z*-axis piezo stage was used for the fine focus adjustment. The microscope system is enclosed in an acrylic box where the temperature is feedback stabilized at 18 °C (*InVivo* Scientific). The stable temperature minimized possible drift caused by temperature fluctuations.

### Excitation system

For excitation of the fluorescent dyes (Cy3/Cy5 and TetraSpeck beads), we used 561 and 643 nm CW DPSS lasers (Cobolt) whose powers were electronically controlled by Acousto-Optic Tunable Filters (Gooch and Housego). The laser beams were spectrally cleaned by a multi-colour excitation filter (Chroma, z488-491/561/640x), reflected on a multi-channel dichroic mirror (Chroma, zt488-491/561/640rpc) and transmitted through the objective lens. We used a TIRF-illuminator to easily and accurately control the TIRF angle for low background noise. We also placed a half-wave plate to make the excitation beams p-polarized, to excite dipoles having a broad range of orientations. Note that the polarization of the excitation beam only affects the brightness but not the shape of the PSF, which is solely determined by its emission dipole orientation.

### Emission system

The emitted fluorescence was collected and collimated by the TIRF objective lens. The collimated light was transmitted through an emission filter (Chroma, zet488-491/561/640 m), which allows transmission of the light only in the range of 580–620 nm or > 660 nm. The beam was directed towards the side-port of the microscope, followed by a C-mount × 2.5 relay lens (Nikon, MQD42120) to achieve a total × 250 magnification, which is sufficiently high to spatially resolve the anisotropic shapes of the diffraction-limited Cy3/Cy5 spots (Fig. 2). We used a Dual-view beam splitter (Cairn Research, Optosplit II) to spectrally split the Cy3 and Cy5 fluorescence. The Dual-view has a dichroic mirror (Chroma, 630 dcxr) that reflects the emission from Cy3 while transmitting the emission from Cy5. Those signals then passed through emission filters (Chroma, HQ610/75M and HQ580/60M for Cy3; HQ680/60M for Cy5). The separated paths were recombined and directed onto different areas on the EMCCD camera for imaging (Supplementary Fig. 2).

### EMCCD camera

For imaging the fluorescent spots (TetraSpeck and Cy3/Cy5), we used a highly sensitive EMCCD camera (Andor, iXon plus), which has a 1,004 × 1,002 pixel array consisting of 8 μm square pixels. The × 250 magnification of the microscope resulted in an effective pixel size of 8 μm/250=32 nm. We used a calibration bar with 2 μm-separated stripes, to confirm this. We used a gain setup (Pre-Gain: × 1, EM-Gain: × 400) that allowed sufficient amplification of the tiny signal from single fluorescent molecules.

### Bright-light illumination

Drift of the emission paths over time was a concern as that would render the mapping calibration between the channels unreliable for use with the single-molecule data recorded at a different time point^5^. To this end, we used a lamp (Nikon, DiaLamp) to bright-field illuminate the imaging channels to be used for the correction of the emission path drift. We adjusted the intensity of the lamp such that the signal was intense without saturating the detector. The transmitted light clearly reveals the imaging windows for the two channels on the camera (Supplementary Fig. 9).

### μ-Manager

We used μ-manager (version 1.4.16) as a main programme to control most of the components in the setup, including microscope, motorized and piezo stages, PFS, EMCCD camera, lasers, Acousto-Optic Tunable Filter and shutters. The μ-manager is a convenient tool to collect data in an easy and a consistent manner. Automated data collection was useful for high-throughput sampling.

### Sample flow-cell chamber

Three flow-cell chambers, respectively, for the dsDNA (20 and 30 bp) and the TetraSpeck beads for the mapping calibration, were made using a PLL-coated coverslip, a microscope slide (Gold Seal Micro Slides), and double-sided tape (Scotch, 3 M). First, 10 μl of 10 pM dsDNA 20 bp/30 bp were flowed into the first/third sample chambers, and 10 μl of 10 pM TetraSpeck beads was flowed into the second sample chamber. Second, they were incubated for 2 min and washed with 50 μl of the buffer to eliminate any weakly attached molecules or beads. Finally, 20 μl of imaging buffer was flowed through the chambers and they were sealed with vacuum grease to prevent drying.

### Data collection

We collected data in the order of dsDNA (30 bp) in chamber 3, dsDNA (20 bp) in chamber 1, and finally TetraSpeck in chamber 2. TetraSpeck beads were sparsely coated on the PLL surface to avoid overlapping beads. The 561 and 643 nm lasers (∼ 2 mW) simultaneously excited the beads and the different colours were imaged on different channels of the EMCCD camera (Supplementary Fig. 2). To obtain a sufficient number of mapping spots covering the entire field of view, multiple sets of beads were imaged as time-lapse movies by repeatedly translating the stage by 50 μm. The time-lapse movies were recorded at 10 Hz sampling rate. The dsDNA data were recorded at different locations as time-lapse movies taken at 2 Hz with simultaneous excitation of the lasers with ∼10 mW power. This was done for both the 30 bp and the 20 bp sample. All measurements were done with the PFS to maintain the same focal depth. For the *post-hoc* correction of the emission path drift, we took ten snapshots of bright-light images right after movies of, respectively, TetraSpeck beads and dsDNA.

### Calibration of map between images in the two channels

To map between the experiment's two channels, we used 100 nm TetraSpeck beads attached non-specifically to the coverslip surface. They imaged near the focus as diffraction-limited spots in both channels of our experiment (Supplementary Fig. 3). In total, we used 93 time-lapse movies each consisting of 90 frames. For each movie, we automatically found the approximate pixel positions of the PSF centres in the first channel using a combination of minimum and maximum filtering of the intensities in the field of view (FOV), which detected local maxima in the image above an intensity threshold. The corresponding centres in the other channel were found using a rough estimate for the distance vector mapping one channel to the other. Subsequently, using these approximate centres as starting estimates, we used unweighted least-squares fitting in conjunction with a 2D Gaussian plus a constant ‘background' (GME)^4^,^23^ to localize the centres of their images in each frame in each channel with nm-precision (Supplementary Figs 3 and 4). We then rejected any bead with centre within 24 pixels of any other bead's centre in the same image. This conservative acceptance criterion qualified 7,324 spots (in both channels) for further analysis.

To eliminate the effect of translational drift of the sample stage, we considered the vector difference between each bead's locations in the two images. These vector separations are unknown *a priori* and fluctuate through a movie, so we calculated their time-averages and standard deviations. To eliminate loosely fixed beads and artefacts from the automatic detection scheme, we accepted a bead for further analysis only if the components of its vector separation between channels fluctuate with normal distributions around their mean values and that with s.d. given theoretically by the number of photons in the diffraction-limited images of the bead^4^ (*P*>0.015, Pearson's chi-squared test for distributions; Supplementary Fig. 4). We also disregarded beads with estimated mean widths that differed from the population average by more than three standard deviations. This eliminated beads that had imaged with overlapping PSFs. These filters left us with 5,622 beads qualified for the map calibration.

We calculated the power spectra of each time series of vector separations. For negligible autocovariance in time, the expected power spectrum is constant and equal to the variance of the estimated separations. By virtue of the central limit theorem, the experimental power spectral values are exponentially distributed around that variance (Supplementary Fig. 5). We found that the power spectra were consistent with the hypothesis of negligible auto-covariance, because the *P*-values (Pearson's chi-squared test for distributions) for this hypothesis found across the population of beads were approximately uniformly distributed in the interval [0,1]. Also, we found no discernable correlations between the equal-time values of the two coordinates of the vector separation, because the *P*-values (Pearson's test for correlation) encountered in the population of beads were uniformly distributed in [0,1]. Therefore, we proceeded to calculate the error on the time-averaged vector separations as s.e.m.=s.d./✓*N*, where *N*=90 is the number of frames in each time-series. Typically, we found an s.d. of ∼6 nm, hence an s.e.m. of ∼0.6 nm. Therefore, although GME is less precise than other methods^4^, it is faster and here sufficiently precise when positions are estimated as long-time averages.

A given point in the FOV was mapped between channels, by linear least-squares fitting two second-degree polynomials in both coordinates—one polynomial for each coordinate, *x* and *y*, mapped—to the mappings of all beads in a circular area of 22 pixels radius surrounding the point (Supplementary Fig. 8). Typically, such an area contained ∼25 beads, which over-determines the second-degree polynomials with its six degrees of freedom. The map determined by two such fitted polynomials does not map the fiducial beads it is based on, onto their actual images. The vector difference between a bead's mapped coordinates and actual coordinates show no spatial correlations; these errors are essentially random. This is presumably because the two relevant dyes have different locations within any given bead. Combined with the random orientation of the beads on the surface, this explains our observed random mapping errors. These errors add to the shot noise errors on bead positions to an error with s.d. ∼ 5 nm (Supplementary Fig. 8). We propagated these errors to calculate the error of the mapped position and found it to be ∼2 nm (Supplementary Fig. 8). We include this mapping error every time we discuss the theoretical error on time-averaged vector distances and Euclidean distances between Cy3 and Cy5 throughout the paper (Figs 3e–g and 4a and Supplementary Fig. 14). The accuracy of the map is verified implicitly (Figs 3 and 4 and Supplementary Fig. 14). Had the spots been imaged significantly away from the focus, one would expect the map between channels to change because of aberrations. Here, however, all variation in distance measurements is accounted for by shot noise, which leaves no room for systematic errors.

### Detection of and correction for drift of emission path

During experiments we occasionally observed drift in both imaging windows with respect to the EMCCD pixel array (Supplementary Fig. 9). This drift was likely due to drift in the macroscopic springs that control the mirrors in the Dual-view and thus the alignment of each channel onto the CCD camera. We measured this drift by using bright illumination to create a clear contrast between the imaging windows and the background (Supplementary Fig. 9) and fitting a model to the resulting images of the windows. The model is a rectangular aperture with diffraction at the edges. This diffraction causes a smooth transition in intensity from bright light inside each rectangle to dark background outside it. This intensity profile we modelled with an error function, which results from using a Gaussian approximation for the point-spread function. We parameterized this model rectangle with a common constant background, total intensity, rotation, width and four location parameters (Supplementary Fig. 9). Typically, the shape and orientation of the rectangle did not change between the time of measurements of dsDNA data and corresponding calibrations of the map, but occasionally we detected translational drift (Supplementary Fig. 9). If this drift was significant, we corrected for it. In practice, the position of a dye in one channel was translated to account for the drift experienced by its imaging window with respect to the position of that imaging window at the time of the map calibration measurements. Then, the corrected dye position was mapped onto the other channel. The position of the other dye was simply translated to account for drift in that channel.

### Calibration of the EMCCD camera

One must know the number of photons in an image of a fluorescent probe in order to calculate the precision with which the probe may be localized. This requires a calibration of the EMCCD camera in order to convert its output signal to its equivalent number of photons. To that end, we first determined the constant offset in the camera's output signal from a dark image obtained with the shutter closed. It was 300. This constant is added by the camera to all signals before they are output, and it should not be confused with a photonic background; it does not fluctuate.

Next, we used two areas of constant photonic background from the two channels, respectively, recorded during dsDNA data recording. In each of these areas, all pixel outputs are drawn from the same distribution, presumably; this is what we mean by constant background: Same statistics in each pixel. For each area, the distributions of pixel outputs was used to calibrate the camera. We used the full EMCCD output signal distribution^4^, which was fitted to the output pixel values, to determine the remaining values of the EMCCD parameters, namely the gain, the width of the Gaussian readout-noise distribution (Supplementary Fig. 15). We found, essentially, identical values in the two areas and proceeded to use the average value everywhere to calibrate the camera. Also, these parameters were used with the full EMCCD signal distribution in the calculation of the likelihood whenever we localized using MLE.

The electron multiplication process in the EMCCD camera effectively results in variances that are inflated by an exact factor of 2 compared with pure shot noise. This is often referred to as excess noise. Throughout the paper, when we refer to shot noise, the correction for this excess noise is implicitly assumed.

### Analysis of fixed fluorophores

We used MLE and the theoretical PSF for an isolated fixed fluorophore located close to the coverslip and imaged (nearly) in focus, using a slightly modified version of the procedure described in ref. 4.

Briefly, we modelled the fluorophore emission as a dipole transition. A complete vector description of the emission was then applied to the refraction at the relevant interfaces in the microscope and diffraction by the objective. Assuming perfect imaging—that is, loss-free light propagation and negligible optical aberrations—we obtained the theoretical PSF. For each probe, we used the average emission wavelength incident on the camera after the various emission filters and dichroics in the emission path were accounted for. We used 580 and 670 nm for Cy3 and Cy5, respectively.

Furthermore, we used an analytical approximation to the PSF of a fixed fluorophore. This approximation was sufficiently accurate and precise for the purpose of estimating location and orientation (Supplementary Figs 10 and 11). The PSF's dependence on focus was included in its analytical description by taking into account the appropriate phase changes^20^ in the electric and magnetic field components in its calculation. For each wavelength and for each value of the defocus in increments of 1 nm in a range of ±250 nm around the focus, we obtained an accurate analytical approximation to the full PSF, proceeding as previously^4^. This tabulation of this analytical approximation allowed us to fit the value of the instantaneous defocus along with the other parameters in each fit.

The MLE was done with Supplementary Software. The present method for simultaneous estimation of location and orientation of an isolated dipole we refer to as diPOLE for brevity. Thus, diPOLE uses MLE based on the true theoretical PSF of an isolated fixed dipole that is more or less in focus, but with the theoretical PSF well-approximated analytically to achieve a dramatic and necessary speedup of computation with essentially no cost in the form of degraded accuracy or precision (Supplementary Figs 10 and 11).

Using this methodology, we found that the effective focal depth in each of the two channels differed slightly; specifically, we found that the Cy3 and the Cy5 fluorophores, respectively, yielded defocuses of −30±40 nm and −70±25 nm (mean±s.d.).

We compared the theoretical PSF (in its accurate analytical approximation) with the experimentally measured intensity distributions of fixed fluorophores in a number of ways. First, we directly compared measured PSFs pixel by pixel to each pixel's expected value (Fig. 2e,f). Discrepancies between measured and expected pixel values were rescaled with their theoretical root mean squared deviation as calculated from the theoretical covariance matrix and assuming photon shot noise to be the only cause of discrepancy. Second, we binned pixels with similar expected values for the light intensity they register. We calculated the mean value for the expected signals of all pixels in a given bin. Against this value, we plotted the mean value and s.e.m. of the actually recorded values for all pixels in the same bin (Fig. 2g,h). Repeating this for the full range of expected pixel values, we expect the resulting points to fall on a straight line through the origin with unit slope for accurate theoretical descriptions of the diffraction-limited patterns. This is clearly our case here. Finally, we assessed the distribution of the rescaled signal fluctuations (Fig. 2e,f). For sufficiently large signals (typically satisfied everywhere because of background fluorescence), such rescaled fluctuations should scatter as a standard normal distribution, for an accurate theoretical PSF (Fig. 2i,j).

Recently, dipoles with not entirely fixed orientations have been studied for the effects on localization of their slack in orientation^18^,^25^. Here, however, we demonstrated near-perfect agreement between the theoretical PSF with fully fixed dipole orientation and the measured intensity distributions (Fig. 2). Although this agreement was compelling, it alone did not guarantee that accurate estimates resulted from applying that PSF to experimental data. We therefore demonstrated that it did, using two differently coloured fluorophores fixed within the well-known structure of dsDNA (Figs 3 and 4). Thus, the PSF described above modelled reality sufficiently well to guarantee robust estimates of orientations and positions of fixed fluorophores.

For distance measurements, we calculated the weighted averages of the vector separation. As weights we used the inverse of the theoretical covariance matrix, assuming shot noise was the only source of variance (Fig. 3a,b,e,f). These averaged vector separations were used to calculate the 2D Euclidean distance between the probes (Fig. 3g).

To estimate the angle between emission dipoles, we calculated the scalar product between their vectors (Fig. 3i). Like the angle between the dipoles, this scalar product is independent of the orientation of the DNA molecule to which the probes are fixed. Consequently, the angle between probe pairs is the same for all DNA molecules, apart from some molecule-to-molecule variation. The noise on these angle estimates is so small compared with the expected value for the angle that the appropriate non-Gaussian distribution of relative angles is well approximated by a Gaussian (Fig. 3i,j and Supplementary Note 1). Thus, the weighted average of the individual relative angles is an unbiased estimate for the mean relative angle and we used that.

To determine the spatial orientation of the dsDNA construct, we required that the tangent vectors of the dsDNA structure agreed with the estimated orientations of the emission dipoles of Cy3 and Cy5, and then we solved numerically for the pitch, yaw and roll angles of the dsDNA (Supplementary Figs 12 and 13). The emission dipoles are not directional, which leads to two solutions. Each of the two predicted structures was then compared with the measured time-averaged values for the vector separation between the probes in units of the theoretical error on that estimate. This allowed us to single out one solution for the dsDNA orientation over the other and associate this solution with an odds value in favour of it (Supplementary Fig. 14).

### Simulations

To verify that diPOLE is both accurate and precise also when we speed up its computations by using an analytical approximation to the true PSF, we generated data based on the full evaluation of the true theoretical PSF for fixed fluorophores and applied diPOLE with the analytical approximation to these synthetic data. Synthetic pixel output values (signal counts) were generated using the full EMCCD output signal distribution. To this end, for each pixel, a Poisson random variable simulated the number of photons incident on the pixel. Then, based on the simulated photon number, an Erlang distributed random number accounted for the electron multiplication process to which we added a Gaussian random number that accounted for readout noise. Finally, a constant offset was added, as in an EMCCD camera. Images generated in this manner were then fitted using diPOLE with our analytical approximation to the PSF. This was done for various polar angles and distances to the design focal plane in order to demonstrate that neither accuracy nor precision is significantly compromised by our use of the analytical approximation to the true PSF (Supplementary Figs 10 and 11).

## Additional information

**How to cite this article:** Mortensen, K. I. *et al.* Optimized measurements of separations and angles between intra-molecular fluorescent markers. *Nat. Commun.* 6:8621 doi: 10.1038/ncomms9621 (2015).

## Supplementary Material

Supplementary Information Supplementary Figures 1-15, Supplementary Table 1, Supplementary Note 1 and Supplementary References

Supplementary Software Python (python.org) code for the maximum likelihood estimation of position and orientation of fixed dipole emitters positioned close to the coverslip and imaged close to the design focal plane onto an EMCCD camera in a TIRF microscopy experiment (Methods). To this end, the theoretical point spread function is fitted to experimental intensity distributions. To speed up fitting, an accurate analytical approximation to the theoretical point spread function is used (Methods, Supplementary Figs. 10 and 11). The maximum likelihood estimates of the parameters are returned along with their theoretical uncertainties. For details, see the readme file enclosed with the Supplementary Software.
