# Supplementary material for: Optimized measurements of separations and angles between intra-molecular fluorescent markers
Source: Nat Commun. 2015 Oct 16;0:8621. doi: 10.1038/ncomms9621 (PMC4634324; doi:10.1038/ncomms9621)
Supplement: Supplementary Information — Supplementary Figures 1-15, Supplementary Table 1, Supplementary Note 1 and Supplementary References [file ncomms9621-s1.pdf]

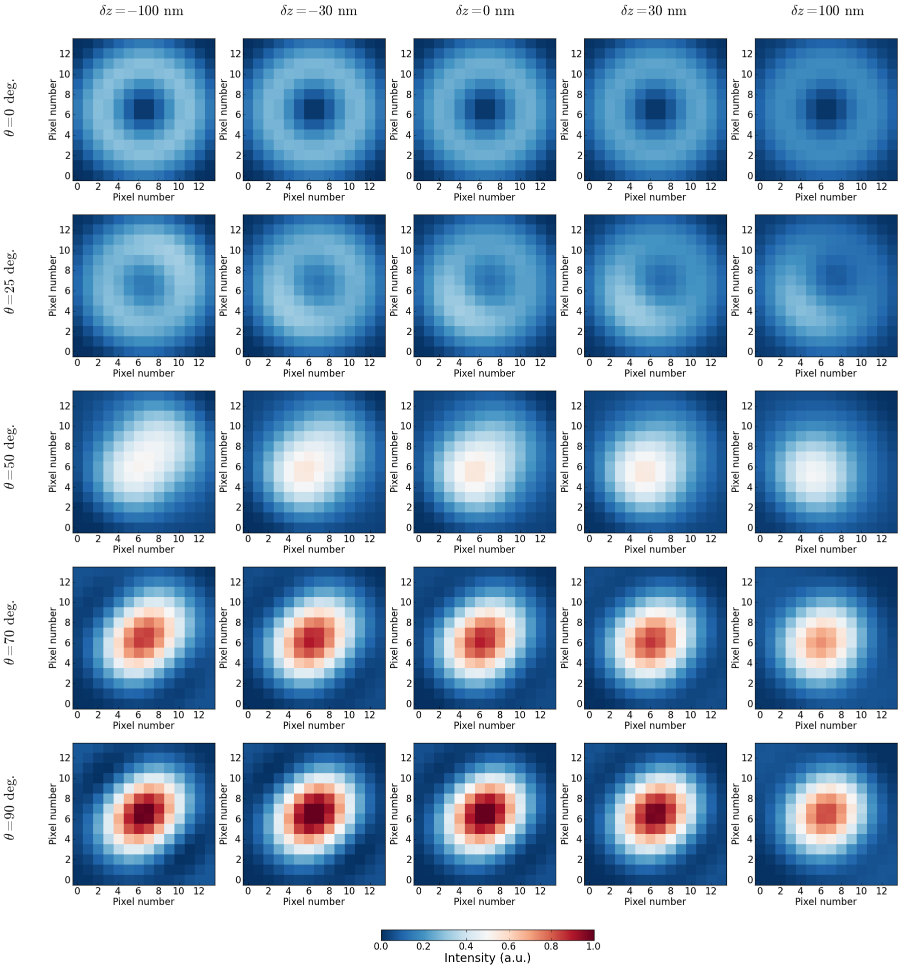

Supplementary Figure 1

Point spread functions for fluorophores with fixed orientations imaged near the focus. The top left panel shows the theoretical intensity distribution from an image of a fluorophore that is fixed in orientation during imaging with azimuthal and polar angles of 225 and 0 degrees, respectively, and imaged at a distance of -100 nm from the focal plane (Methods). The fluorophore's position coincides with the center of the pixel array. We used an emission wavelength of 570 nm and 32 nm square pixels throughout, while the remaining parameters are set identical to the ones used in the analysis of the experimental dsDNA data (Methods). Other panels show the intensity distribution for fluorophores oriented at different polar angles (rows) and imaged at different focal depths (columns). The intensity scale is identical in all panels and given by the color bar at the bottom.

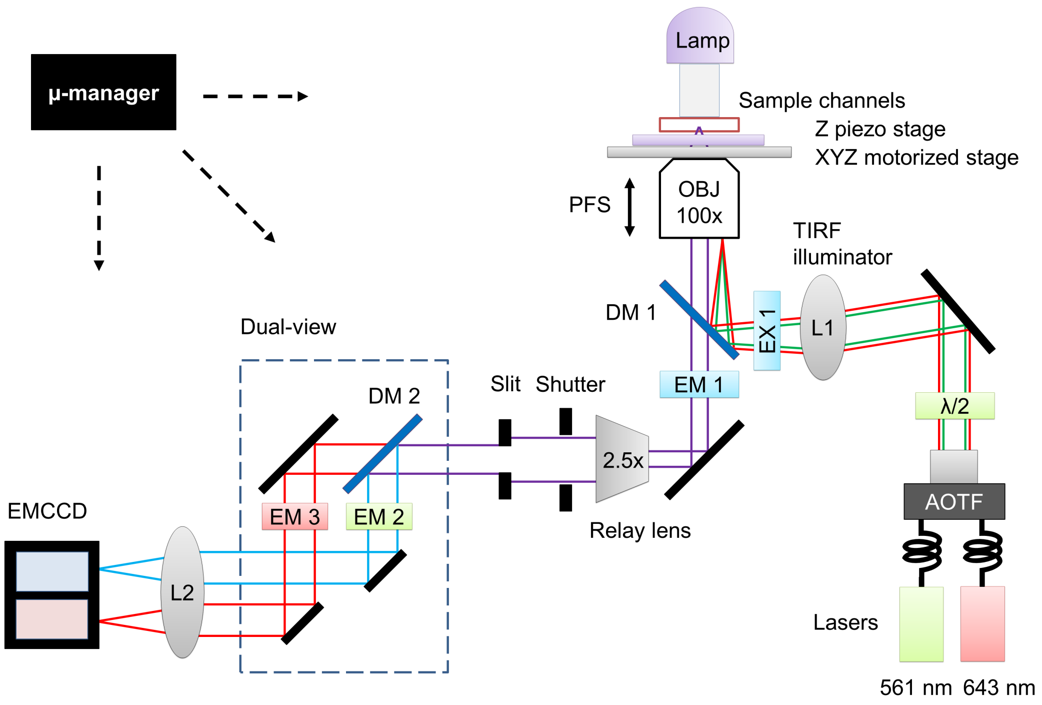

Supplementary Figure 2

Experimental setup. Also see Methods. We used a commercial TIRF scope (Nikon, Eclipse TE2000-PSF) with an oil immersion TIRF objective lens (Nikon, Apo-TIRF 100x, NA 1.49). The Perfect Focus System (PFS) was used throughout to minimize the focus drift. The sample slide was firmly mounted on stages (XYZ motorized and Z piezo stage). 561 nm and 643 nm lasers were coupled and their power was modulated by the Acousto-Optic Tunable Filters (AOTF). The laser beams were focused at the back-focal plane of the objective lens and the angle was controlled by the TIRF illuminator for TIRF excitation of the sample with low background noise. The fluorescence emission was filtered by an emission filter (EM1), expanded by a 2.5x relay lens for increased magnification, and split in the Dual-view (Cairn Research, Optosplit II) where the different emission spectra were split on a dichroic mirror (DM2) and each signal was further filtered by emission filters (EM2 and EM3). Finally, they were imaged on different area of the EMCCD camera (Andor, iXON plus). Bright light from the lamp (Nikon, DiaLamp) was transmitted through the objective lens, split in the dual-view, and imaged on the EMCCD camera whose high-contrast bright image was used to correct for emission path drift at the time of data analysis.

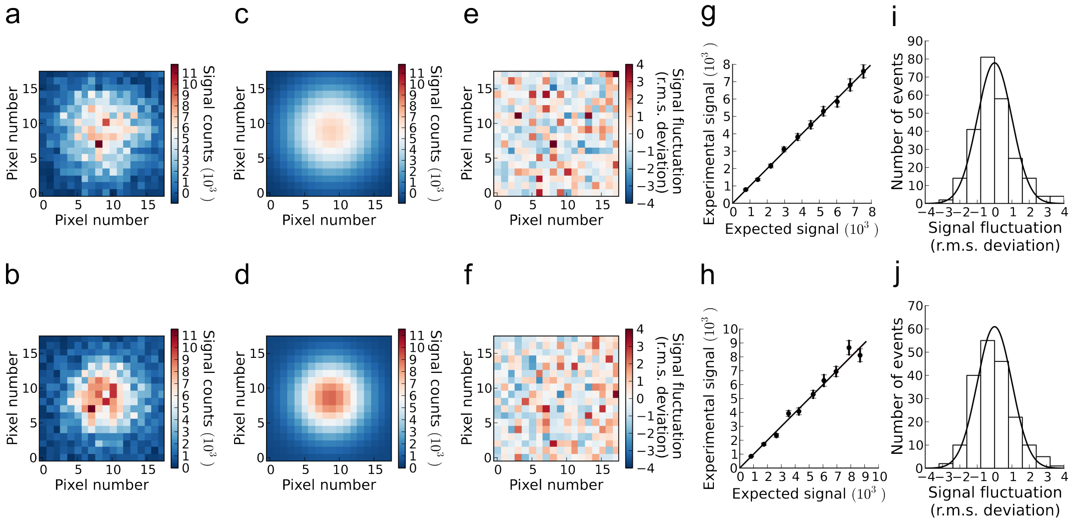

Supplementary Figure 3

Localization of fluorescent beads used as fiducial markers in order to map between the two channels in the experiment. (**a**,**b**) TIRF microscopy images of, respectively, orange and dark red emissions of the same multi-colored fluorescent bead (Methods). (**c**,**d**) Theoretical images with parameter values obtained speedily with GME, i.e., least squares fitting with equal statistical weights for all pixel-signals in the fit, in conjunction with a 2D Gaussian-plus-a-constant PSF[1]. (**e**,**f**) Rescaled residuals. Each pixel shows the difference between measured and expected pixel signal values in units of its theoretical r.m.s. deviation as given by the color bar. The theoretical r.m.s. deviation was obtained using Eq. 6 in Ref. 1 with the parameters fitted in **c** and **d**. (**g**,**h**) Measured signal values compared to expected values. We binned the expected signals and their associated pixels with experimental values according to the expected values. For each bin, the mean experimental signal is plotted against the expected signal with an error bar indicating the theoretical s.e.m. Data points shown should scatter about the straight line through the origin with unit slope, each in a Gaussian manner with s.d. equal to the shown error bar. (**i**,**j**) Histograms of rescaled residuals. For sufficiently large expected pixel signals, theory predicts a normal distribution with zero mean and unit s.d. (solid line) for the fluctuations in **e** as well as in **f**.

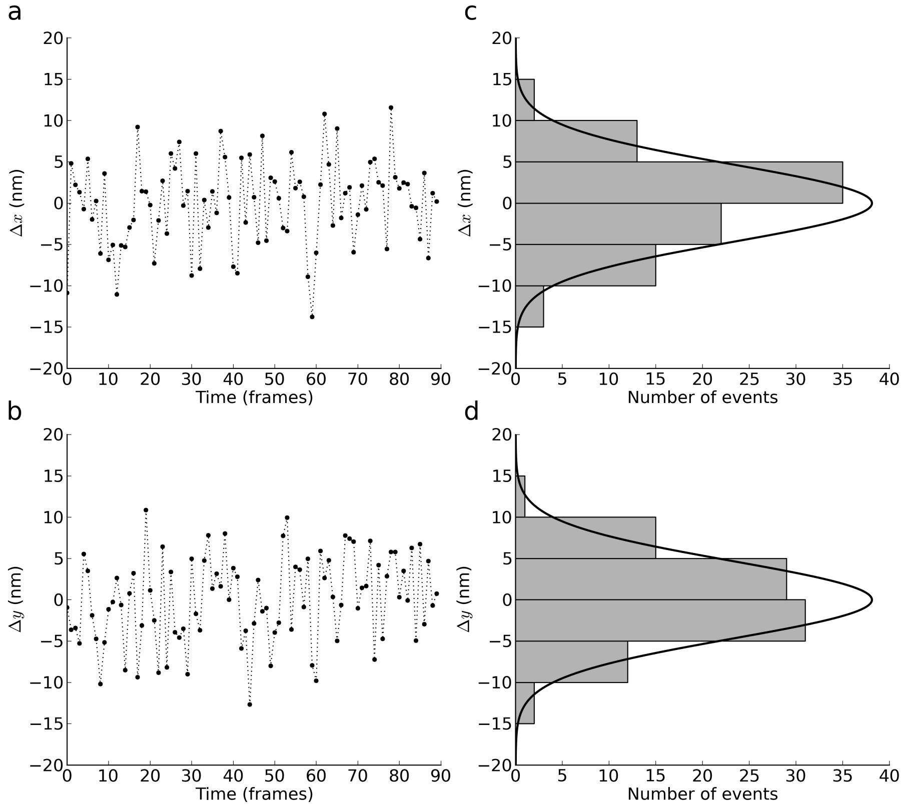

Supplementary Figure 4

Time series of fluctuations in the vector distance in the image plane between two spectrally separated images of the same fluorescent bead. (**a**) Using GME, we localized the center of the image of a single fluorescent bead in both channels for each frame in a time lapse movie consisting of 90 frames (Methods, Supplementary Fig. 3). For each frame, we computed the separation between such images along the *x*-axis and subtracted its time-averaged value. The resulting residuals are shown here (connected black dots). (**b**) Same as **a** for the separation along the *y*-axis. (**c**) Histogram of the residuals from **a** overlaid with a normal distribution centered on the origin and with s.d. equal to the theoretical s.d. for the estimator of separations (solid line), computed using parameters from the analysis in Supplementary Fig. 3. The distribution of residuals of measured separations is consistent with the theoretical expectation with $p = 0.12$. This p-value was found using Pearson's chi-squared test for the distribution. (**d**) Same as **c** for the residuals from **b**. Here we found consistency with $p = 0.16$. We used a bead as fiducial marker only if the fluctuations in the vector separation between its dark red and orange images were consistent with photon shot noise with $p > 0.015$ for both coordinates.

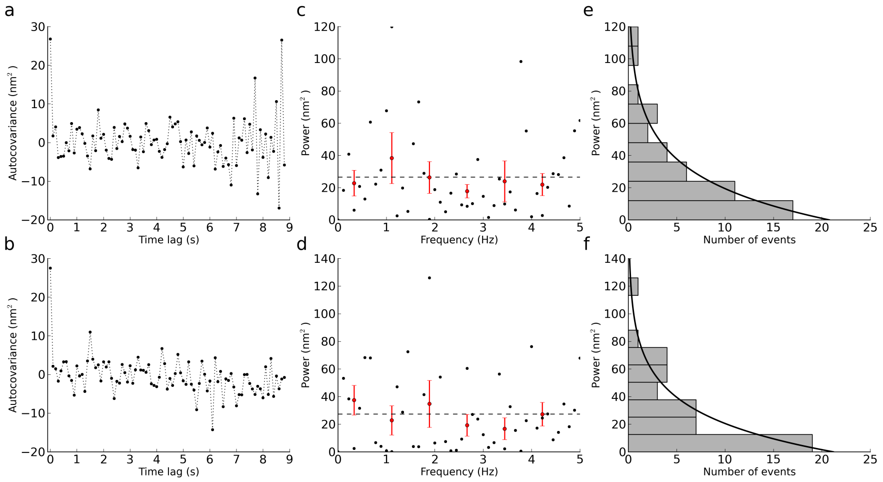

Supplementary Figure 5

Fluctuations in the vector separation between a bead's images in the two channels has negligible autocovariance in time. (**a**) From the residuals of separations between a bead's orange and dark red images along the *x*-axis from Supplementary Figure 4a, we calculated the autocovariance (connected black dots). Its value at zero lag is the variance of the residuals. (**b**) Same as **a** for the separations along the *y*-axis from Supplementary Figure 4b. (**c**) The power spectral values of the residuals in **a** (black dots). Uncorrelated noise gives rise to a uniform ("white") power spectrum with value equal to the variance of the signal (dashed line). Block averages of the power spectral values (red points) are consistent with such white noise. (**d**) Same as **c** for the input data from **b**. (**e**) Histogram of power spectral values from **c**. These values are independent and identically exponentially distributed with mean value equal to the variance of residues (solid line), if they originate in white noise with finite variance (Methods). The measured distribution is consistent with this theoretical expectation with $p = 0.95$. This *p*-value was found using Pearson's chi-squared test for the distribution. Consequently, due to the absence of temporal correlations, the time-averaged separation has s.e.m. = s.d./$\sqrt{N}$. Here $N = 90$ is the number of frames in the movie and s.d. = 5.1 nm, so s.e.m. = 0.5 nm. Thus our estimates for the vector difference between the two images of a bead have sub-nanometer stochastic errors. (**f**) Same as **e** for the power spectral values in **d**. Here we found $p = 0.55$ and s.e.m. = 0.6 nm.

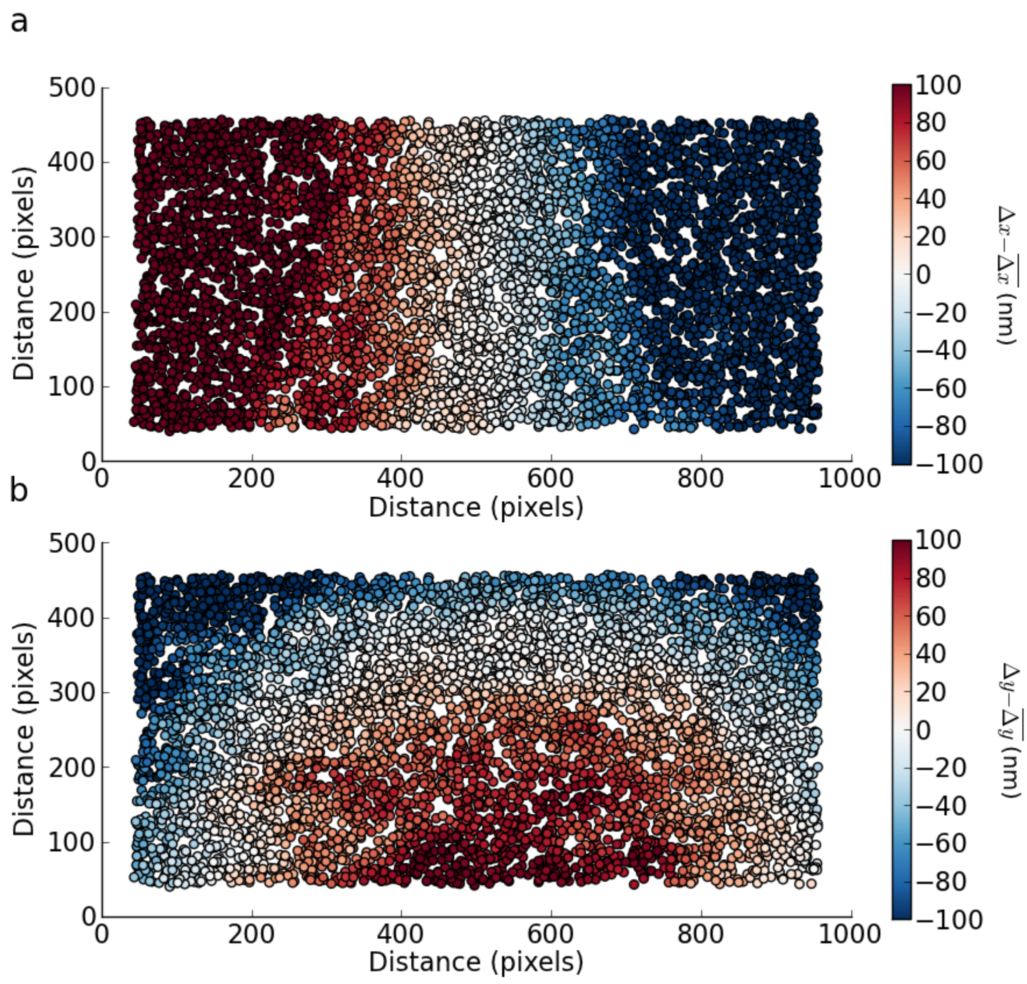

Supplementary Figure 6

Map of fiducials between channels. (**a**) We estimated the time-averaged positions of 5622 beads in Channel 1 as well as in Channel 2 as described in Supplementary Figs. 3-5. For each bead, its average position in the field of view is plotted (colored points), and the point's color indicates the extent to which the difference in *x*-coordinate between the point's coordinates in the two channels differs from the population average of the same quantity. The effective pixel size was 32 nm. (**b**) Same as **a** for the difference in *y*-coordinates. Panels **a** and **b** show that the map between channels measured experimentally with fiducial beads is not a constant vector translation when measured with the precision we need: On top of the constant vector translation we see smooth spatial variations, as expected from optics, and superposed on that, a speckled pattern with a characteristic length scale comparable to the diffraction limit (Supplementary Fig. 7).

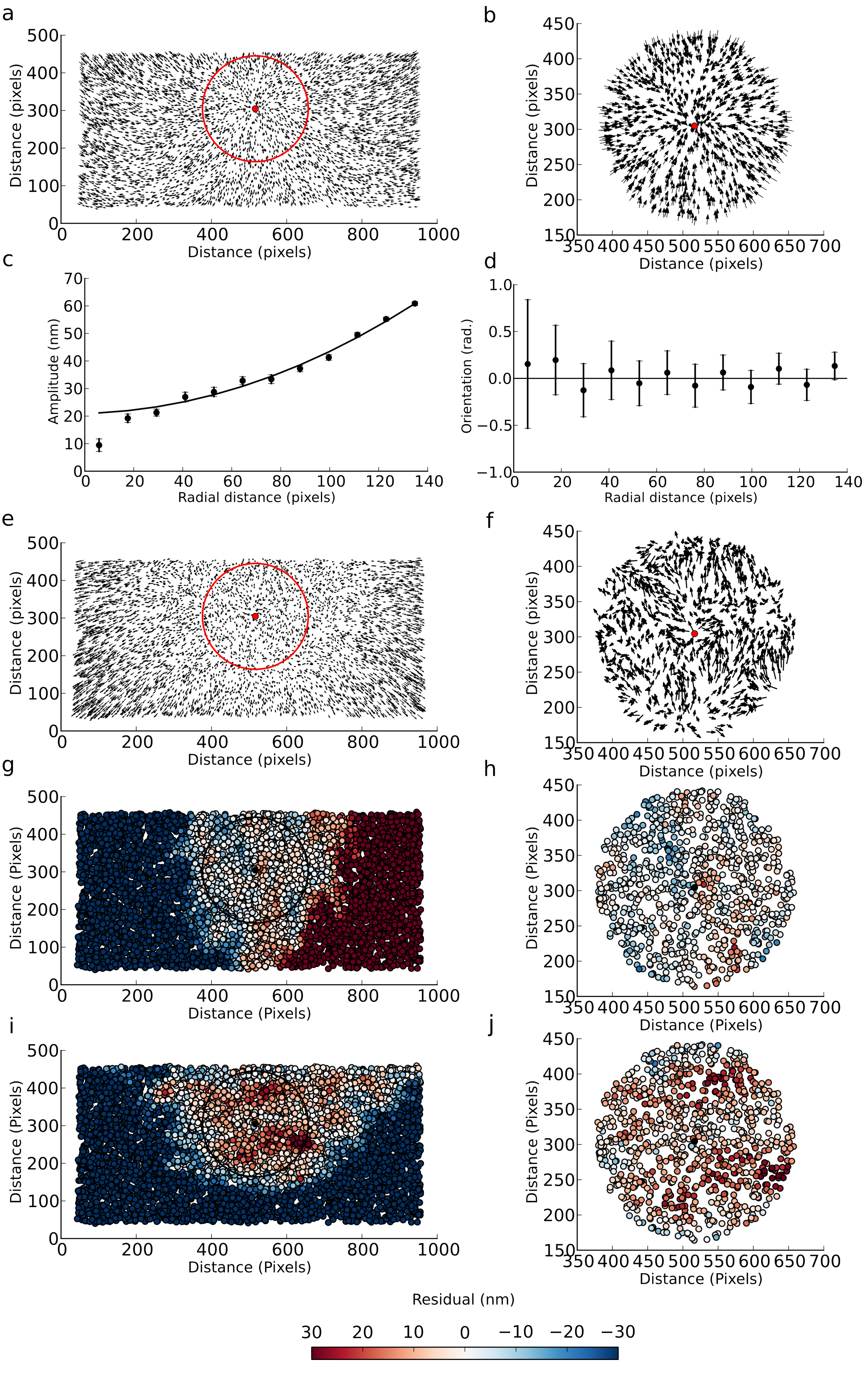
a
b
c
d
e
f
g
h
i
j
Distance (pixels)
Distance (Pixels)
Amplitude (nm)
Radial distance (pixels)
Orientation (rad.)
Residual (nm)

Supplementary Figure 7

Analysis of the experimentally recorded map between channels reveals a speckled pattern that makes a global map between channels impossible. The map between channels that we observed with fiducials, is not just a constant translation vector, but differs from that in a manner that depends on location in the image plane (Supplementary Fig. 6). Much of these aberrations we could explain as (similar to) spherical and chromatic aberrations: They are radial displacements relatively to the optical axis and depend only on the distance from it, and hence much can be captured by a calibrated global map between channels. This figure shows, however, that too much of the actual map remains undescribed by a global map. (**a**) We estimated the position of the optical axis (red point) in the field-of-view (FOV) as the position that best explains the observed map between channels as a fixed displacement plus a radial aberration. This was done by minimizing local deviations from this explanation, the sum of all squared angles between observed aberrations (Supplementary Fig. 6) and the radial direction. The result defines a translation vector, the vector that maps the optical axis in one channel to the optical axis in the other channel, plus observed aberrations from this constant map, shown as arrows at the position of each bead. (The effective pixel size was 32 nm.) (**b**) Blow-up of the area in **a** enclosed by a red circle there. In this vicinity of the optical axis, these arrows all point towards the optical axis (red point). We grouped all translation vectors in this area into bins according to their distance to the optical axis. (**c**) For each such bin we calculated the mean amplitude of the translation vectors (black dots) and plotted them as a function of the bin's distance to the optical axis. Error bars represent s.e.m. A second-degree polynomial fits the data (solid line). (**d**) For each bin, we also calculated the mean of angles between aberrations and the radial direction. Error bars are s.e.m. The values scatter around zero, as they would for perfect optics. (**e**) From each aberration in the FOV, we subtracted a radial vector with magnitude given by the fitted curve in **b**. If this curve gives a complete description of observed aberrations, the resulting vector residuals (black arrows) should represent noise, i.e., have uncorrelated random orientations and amplitudes. (**f**) Blow-up of the area in **e** enclosed by a red circle there. Notice that the arrows, however, are correlated on short length scales. (**g**) The *x*-components of the resulting vector residuals from **e** (colored points). The value of each residual is given by the color bar at the bottom of the figure. The optical axis is indicated (black point). (**h**) Blow-up of the area in **g** enclosed by a black circle there and corresponding to the area in **f**. Even in this area around the optical axis, which was used to obtain the model, a speckled pattern of correlations between residuals is revealed. They do not represent noise, but presumably imperfections in the optics. These speckles have a characteristic size comparable to the diffraction limit, which suggests imperfections on a scale smaller than that. The residuals are too large in magnitude to be ignored because of the resolution required for the single-molecule measurements done in the present work. Thus, a global map between channels is not possible. Instead we calibrated local maps (Supplementary Fig. 8). (**i**) Same as **g** for the *y*-components of the resulting vector residuals from **e**. (**j**) Same as **h** for the resulting vector residuals from **i**.

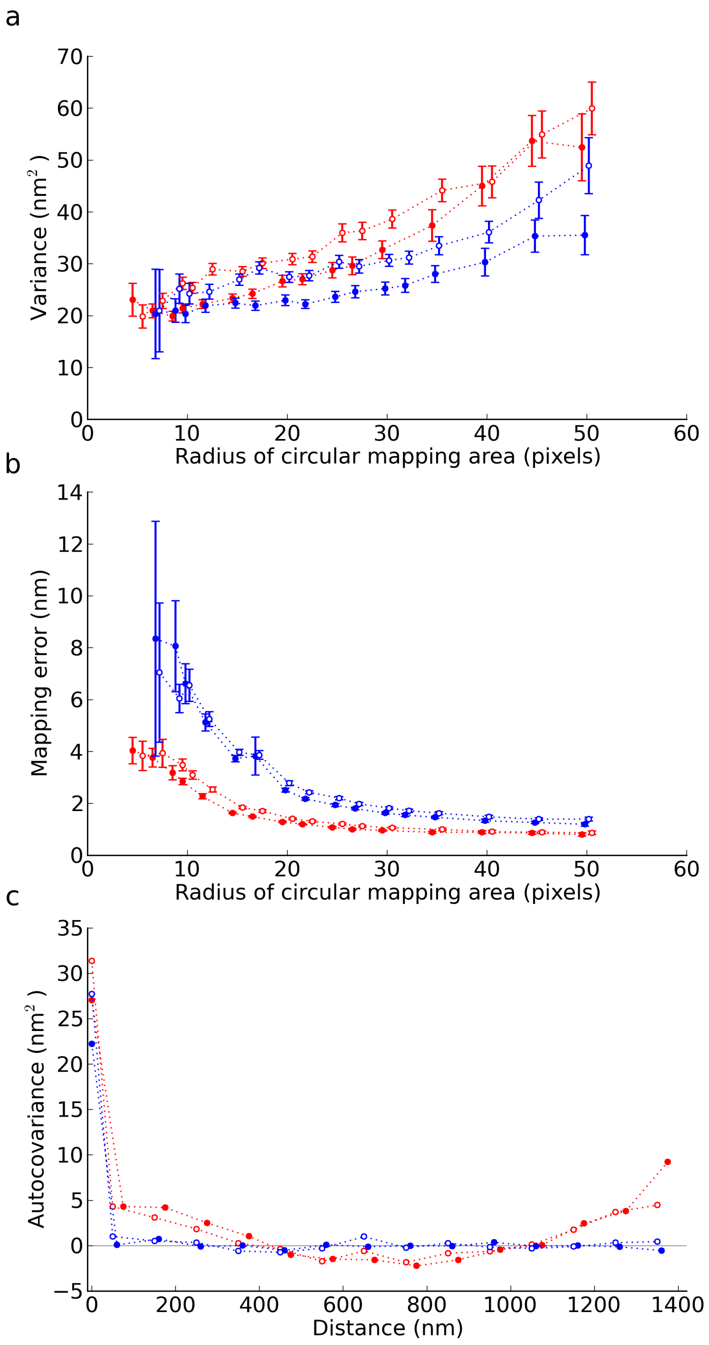

Supplementary Figure 8

Properties of the local map calibration using fluorescent beads as fiducial markers. (**a**) Ability of locally calibrated maps to capture the spatial variation in the global map. We used a locally determined low-degree polynomial to map each coordinate of a position of interest (POI) to its corresponding

position in the other channel (Methods). We used a circular region around the POI and calibrated the map using all accepted beads in this area as fiducials. The polynomial coefficients of the local map were determined with a linear least-squares fit. We divided the imaging region (Supplementary Fig. 6) into mapping areas of different sizes. For each size of mapping area, we determined, respectively, a first-degree (red) and second-degree (blue) polynomial in the *x*- (filled circles) and *y*-coordinate (open circles). We calculated the variance of the residuals between the mapped and the measured positions of the beads in the target channel, where we accounted for the fit of the map and the number of beads included in the fit. Because the number of beads in an area exceeds the degrees of freedom in the polynomial, the quality of a fitted map is quantified by this variance, and this value vanishes only when all bead coordinates from one channel is mapped perfectly onto their measured coordinates in the other channel. Smaller mapping areas lead to lower variance, because the polynomial here is a better approximation of the actual spatial variation in the map. However, below radii of ~20 pixels for the second degree polynomial, the residual variance is constant, indicating a contribution to the variance, which is independent of the area size, most likely the fact that the two dyes have different centers in our fiducial beads. Notice that the second-degree polynomial consistently outperforms the first-degree polynomial for radii larger than ~10 pixels. The effective pixel size was 32 nm. (**b**) Mapping error for the POI. For each map in **a**, we calculated the mapping error for the POI by propagating the variances for each bead as estimated in **a**. To this end we assumed that the residuals of the beads in a map are spatially uncorrelated. This is the case only when a map captures essentially all spatial variation, ensured by using a sufficiently small mapping area (the plateau in **a**). On the other hand, the mapping error decreases with the number of beads included in the map. Therefore, the optimal mapping area balances these two effects. We used a radius of 22 pixels with the second-degree polynomial, which amounts to a mapping error of ~2 nm. We proceed to show that this area is sufficiently small to ensure uncorrelated residuals. (**c**) Autocovariance in space of the mapping residuals for a circular mapping area of radius of 22 pixels. To validate that the map captures essentially all spatial variation, we calculated the autocovariance function of the residuals by binning beads by their Euclidean distance to each other. We bias-corrected the autocovariance to take into account the polynomial fitting and the number of beads contributing to a map. Then we averaged the autocovariance over all maps. The autocovariance of the first-degree polynomial (connected red circles) shows positive correlation for short distances, which demonstrates that the first-degree polynomial is incapable of capturing all spatial variation. The autocovariance of the second-degree polynomial (connected blue circles) is essentially equal to a delta function, hence shows no spatial correlations. This demonstrates the validity of the calculated mapping error in **b** for this size of mapping area using a second-degree polynomial.

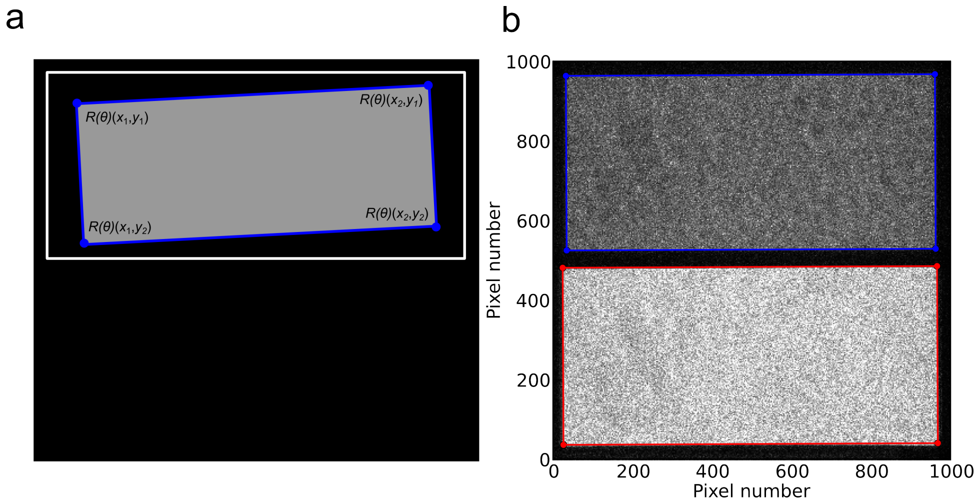

Supplementary Figure 9

Detection and correction of drift in the optical components in the emission path of the microscope. To quantify such drift with nanometer precision, we shone bright light through the microscope. This revealed the imaging windows clearly for both channels. (**a**) We measured the position and orientation of each of these imaging windows by fitting its light distribution with a rectangular area in which diffraction at the edges is accounted for (Methods). To this end, we fitted the rectangle's (blue) position coordinates of its edges (x_1,y_1,x_2,y_2) and its rotation (θ) with respect to the camera pixel array (white rectangle), along with the intensity inside and outside the imaging window. The standard rotation matrix in two dimensions $R(\theta)$ rotates the rectangle into position. Here, rotation with respect to the white reference rectangle is exaggerated for clarity. (**b**) Fits of the imaging windows for the two channels (blue and red rectangles) for a single image are shown. We repeated this for ten consecutive images. Then we compared the fitted parameters for such measurements done immediately after the collection of dsDNA data and the map calibration. Typically, we found no significant change in the rotation of the imaging windows (Student's *t*-test), between these time points. The edge coordinate differences between two experiments were computed for each of the four position coordinates. In all cases, the change in distances for the right and left edges, respectively top and bottom edges, were consistent with each other (Student's *t*-test), which demonstrates that the entire window moves without changing its shape. Consequently, we averaged the distances moved by the right and left edges, respectively top and bottom edges, to quantify the drift of the imaging windows along each of the two axes. Typically, such distances were estimated with an s.e.m. of ~0.5 nm. We corrected for drift (Methods), if the estimated edge distances exceeded twice their s.e.m. In this way, we detected no significant drift in the emission path between the time points of 20 bp dsDNA data collection and the mapping calibration. Between the time points of 30 bp dsDNA data collection and the mapping calibration, however, we detected drift magnitudes of 1.9 ± 0.6 nm and 1.6 ± 0.6 nm, respectively, for the *x*- and *y*-coordinates of Channel 1 and 1.3 ± 0.3 nm and 1.3 ± 0.3 nm, respectively, for the *x*- and *y*-coordinates of Channel 2.

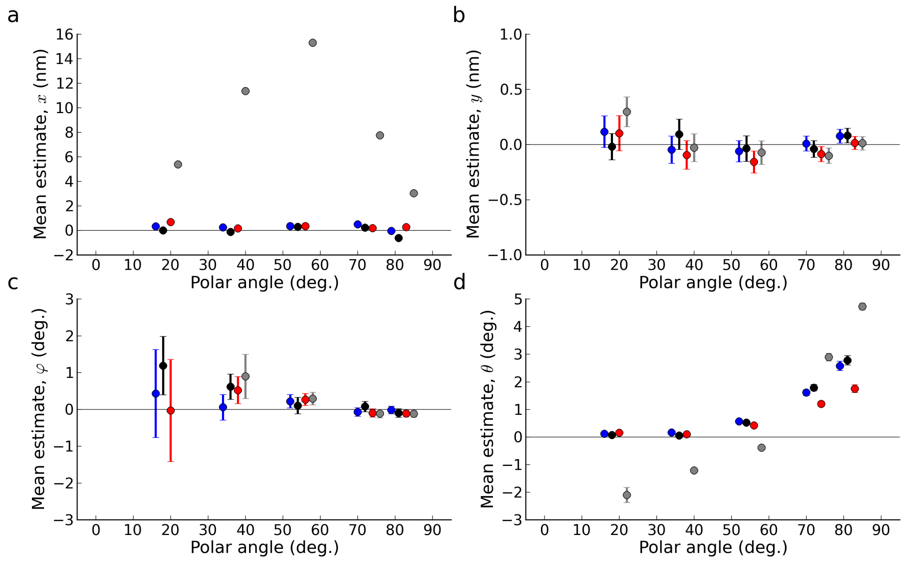

Supplementary Figure 10

Accuracy of diPOLE used with the analytical approximation to the true PSF. We simulated images of isolated, fixed fluorophores for a range of polar angles and distances from focus using the exact expression for the true PSF. Without loss of generality, the azimuthal angle was set to zero. We used physical parameters and EMCCD parameters identical to those of our experiment (Methods, Supplementary Fig. 15). For each image, we first generated a Poisson random variable for each pixel in a pixel array of 14 x 14 square pixels, each with a width of 32 nm. This modeled the photon shot noise using a total expected photon number of 10,000 and a background density of five expected photons per pixel. For each pixel, an output value from the EMCCD signal distribution was simulated using the Erlang distribution, readout noise was subsequently added as a Gaussian random variable, and the resulting signal was offset. For each value of the polar angle and for defocus of 0 nm (black), -30 nm (blue), and -65 nm (red), 500 images were generated. We fitted these images with diPOLE using the analytical approximation for the PSF. To demonstrate the importance of the defocus as a fitting parameter, we also fitted the images for defocus of -65 nm using diPOLE where we (incorrectly) assumed perfect focus (gray). (**a**) Mean estimates for the dipole's *x*-coordinate relative to its true value. The error bars indicate s.e.m. calculated from the estimated values. The mean estimated values with the defocus as a fitting parameter are within a nanometer of the true value (solid line), while the assumption of perfect focus is associated with more than 10 nm inaccuracies for intermediate polar angles. (**b**) Same as **a** for the dipole's *y*-coordinate. The estimates of this coordinate are unbiased, due to the choice of azimuthal angle of the probe. (**c**) Same as **a** for the azimuthal angle. The estimates are essentially unbiased. (**d**) Same as **a** for the polar angle. The average estimates with the defocus as a fitting parameter are all within 3 degrees of the true value for the polar angle. These small biases may be avoided by using the full evaluation of the exact PSF, but at the cost of speed. For most purposes, such small biases are immaterial, however.

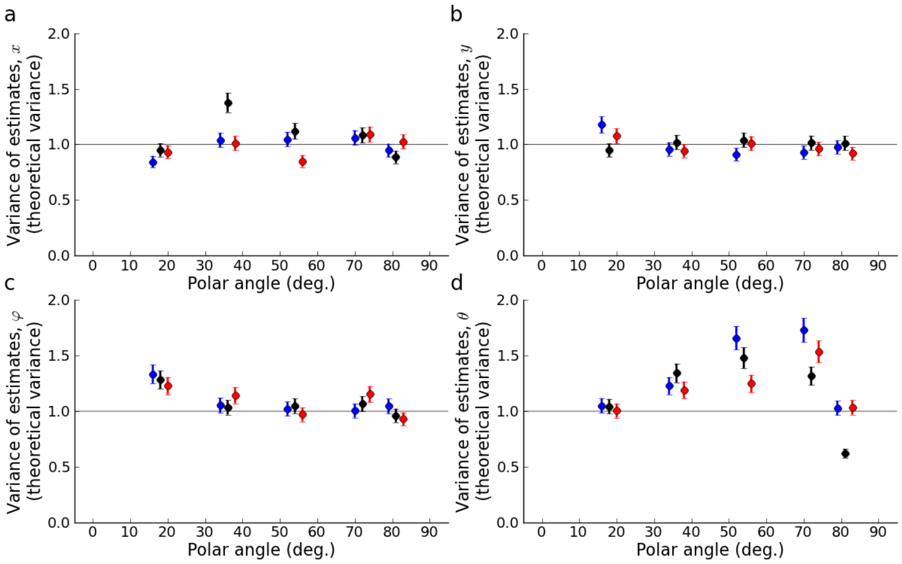

Supplementary Figure 11

Precision of diPOLE used with the analytical approximation to the true PSF. From the simulated images described above (Supplementary Fig. 10), we calculated the sample variance of estimates. (**a**) The sample variance of estimated *x*-coordinates of dipoles in units of the theoretically predicted variance given by the inverse Fisher information matrix for photon shot noise, multiplied by two to account for excess noise (Ref. 1). Error bars indicate the error associated with the estimate of the sample variance. The variances agree with their theoretically predicted values, which represents the lowest possible variance for an estimator (solid line). (**b**) Same as **a** for the estimated *y*-coordinates of dipoles. (**c**) Same as **a** for the estimated azimuthal angles. Variances achieve the information limit for most polar angles. At low polar angles, however, we expect excess variance, because the azimuthal angle becomes ill defined there. (**d**) Same as **a** for the estimates of the polar angle. Here the estimates typically have variances that are larger than the theoretical lower bound, but they remain within a factor of two of it. This finding is consistent with the estimates of the angle between the two probes: they scatter slightly more than the theoretical lower limit on their variance.

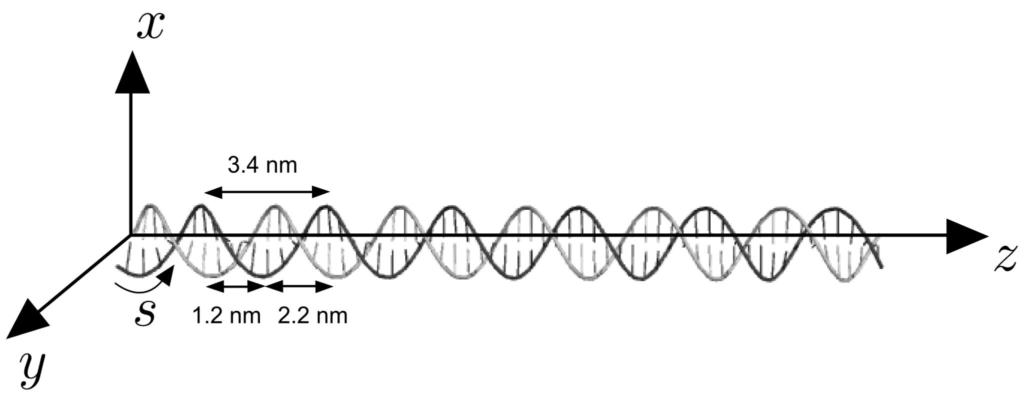

Supplementary Figure 12

Geometric model of dsDNA. In our sample (Methods, Fig. 1a and Supplementary Table 1), each fluorophore (Cy3 and Cy5) is covalently attached to the sugar backbone at two sites, with known separation between them, 20 bp or 30 bp. This integer multiple of 10 bp, one helix turn, ensures that the fluorophore dipoles have approximately identical polar angles. We modeled the B-form dsDNA as a simple double helix with a pitch of 3.4 nm distributed with 1.2 nm and 2.2 nm on the minor and major grooves, respectably. Simulations[2] have shown that the Cy3 and Cy5, both of which replace part of the sugar backbone and bases (Fig. 1a), may interact with the dsDNA located either in a minor or a major groove due to, presumably, hydrophobic effects. So we assumed that each fluorophore hinges with a radius of 1 nm and in a manner that keeps it parallel to the piece of helix backbone it replaces. This makes the relative angle between the two dipoles constant and equal to 74 degrees. We also assumed that the dsDNA was approximately parallel to the coverslip surface. Due to the possible minor/major-groove interactions of the probes with the dsDNA, the distances between them may vary with $\pm$4 bp (dotted lines, Fig. 4a), corresponding to both probes interacting with the closest minor groove, respectively both dyes interacting with the closest major groove. The distance between the probes changes only negligibly for combinations of major/minor groove interactions.

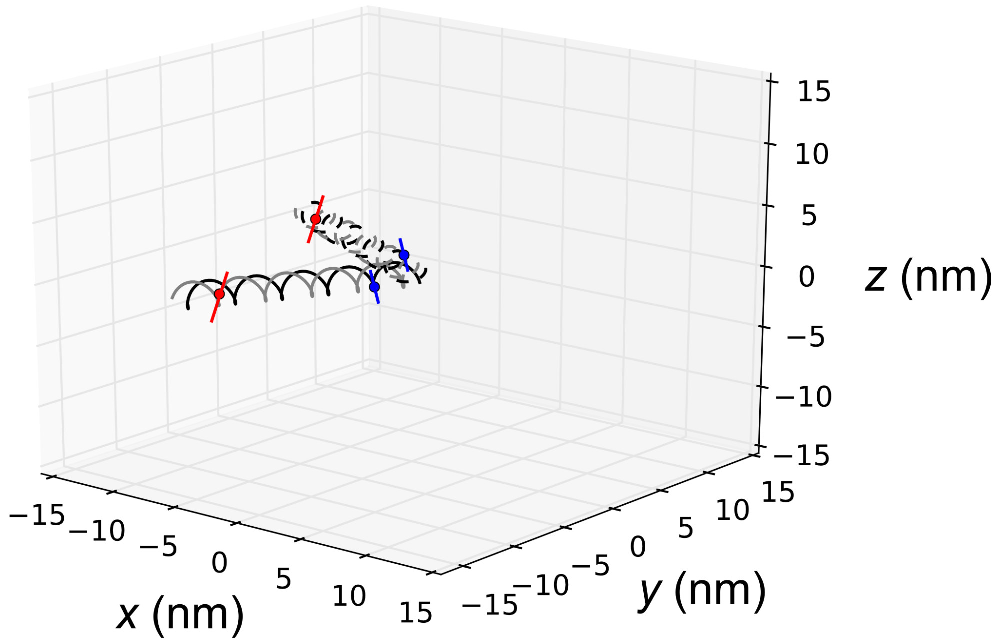

Supplementary Figure 13

Fluorophore orientations suggest two possible orientations for each dsDNA. Given the orientations in space of the two emission dipoles, we still do not know the orientation in space of our dsDNA construct: Two are possible. An emission dipole moment (Cy3, blue line segments; Cy5, red line segments) determines a direction in space only modulo π, i.e., a direction and its opposite direction are the same thing when describing a dipole's orientation. Consequently, knowledge of the polar and azimuthal angles (modulo π) for both probes is not sufficient to determine the orientation in space of the dsDNA construct they are part of. Thus, two different orientations of this construct have the same probe angles, as illustrated here with the probe angles estimated for the molecule discussed in Fig. 3**.** Here, the two strands of each dsDNA are shown in black and gray, respectively, and the two orientations of the DNA are shown with full and dashed lines, respectively. (The probes are separated by 30 bp, and only 50 bp of the full construct is shown to avoid clutter.) These two different orientations of the construct have different angles with the optical axis, however, so the vector differences in position between the probes projected onto the *xy*-plane are different. We compare these to the measured vector differences (Fig. 3e,f), and this comparison uniquely favors one solution (black arrows in Fig. 3e,f; dsDNA with full lines here) over the other (white arrows in Fig. 3e,f; dsDNA with dashed lines here).

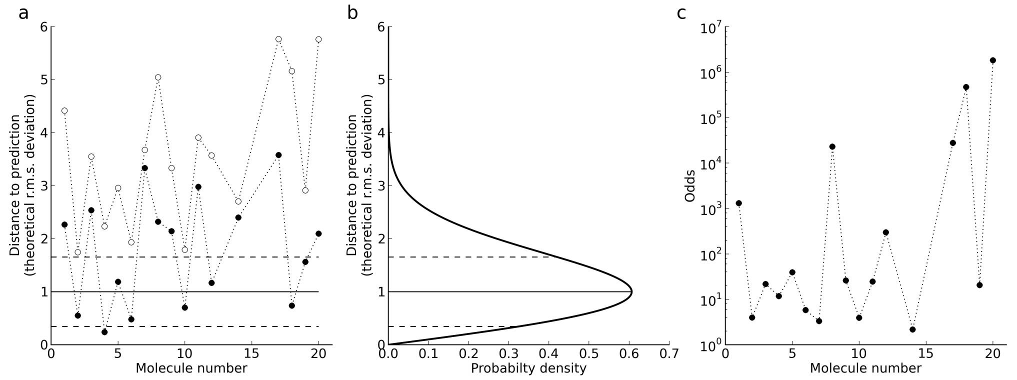

Supplementary Figure 14

Comparison of two possible orientations for each dsDNA molecule. (**a**) We determined two possible 3D orientations of each dsDNA molecule based on estimated fluorophore orientations (Supplementary Fig. 13). Each of the two possible orientations predicts a value for the vector separation of the probes in the *xy*-plane (Fig. 3, Supplementary Fig. 13). We subtracted this value from the measured time-averaged vector separation determined from the fluorophore positions. We then calculated the magnitude of this vector in units of its theoretical r.m.s. deviation, assuming shot noise on the measured positions and mapping error as the only sources of variation. (Thus, in this rescaling, we tentatively ignored contributions from possible minor/major groove interactions and shot noise on measured probe orientations.) For each candidate orientation, this quantified the agreement of the time-averaged vector separations with that orientation. The most probable orientation (black circles) thus has a shorter distance to the prediction than the least probable orientation (open circles). Of the 20 molecules analyzed in this paper, we here included 17 that yielded at least one candidate orientation within 4 units of the theoretical r.m.s. deviation of the prediction. For the remaining three, presumably, dye/surface and dye/dsDNA interactions impede agreement between predicted dsDNA orientations and measured time-averaged vector separations. Notice that this did not compromise distance measurements (Fig. 4a). (**b**) If all variation between time-averaged vector separations and the predictions from the most probable orientation is accounted for, the rescaled distances follow a standard normal distribution on the radial axis (solid line). The mean $\pm$ s.d. (full $\pm$ dashed lines) is indicated. The agreement with data (black circles in **a**) is reasonable. (**c**) To quantify the statistical uncertainty in selecting one orientation over another, we calculated the odds (black circles) in favor of the most probable orientation. This was calculated as (probability to find a rescaled distance to the most probable prediction, which is equal to or worse than the observed, by chance)/ (probability to find a rescaled distance to the least probable prediction, which is equal to or worse than the observed, by chance). The probabilities were calculated by integration of the theoretical probability density in **b**.

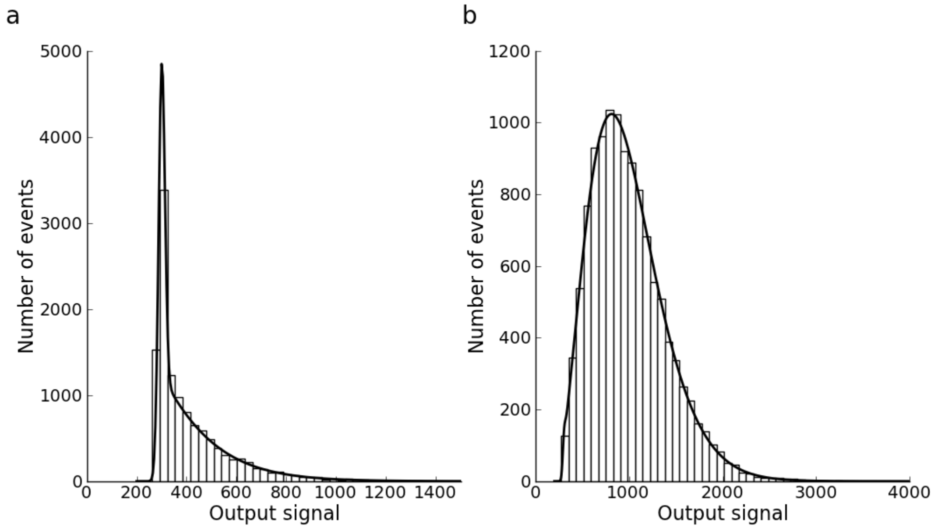

Supplementary Figure 15

Calibration of the EMCCD camera. (**a**) The output signals from an area of 20x20 pixels of uniform background imaged for 30 frames were combined into a histogram. The uniform and constant illumination across all pixels ensured that all pixels' output signals were drawn from the same probability distribution. Non-uniform response statistics for the pixels in EMCCD cameras has previously been reported (Ref. 3), but the present camera (Methods) showed a perfectly uniform response, presumably, due to improvements in designs of EMCCD cameras. Using MLE, we fitted the parameters of the full distribution of EMCCD signal output (solid line) to our calibration set of output signals (Ref. 1). We assumed that the constant offset of the camera was S_{offset} = 300, as previously determined from a dark image. Doing this, we determined the gain of the camera, G = 111. It is the factor that converts between signal output and photon numbers after subtraction of S_{offset}. The same fit also determined the width of the Gaussian distribution readout noise $\sigma_{\text{noise}} = 13$, and the expected number of photons incident on each pixel in one frame, $\nu = 1$. (**b**) Same as **a** using a different area of the EMCCD camera with higher constant background. Here, we found parameter values of G = 114, $\sigma_{\text{noise}} = 14$, and $\nu = 6$. The results for the gain from **a** and **b**, respectively, are within a few percent of each other and we proceeded to use their average value to convert between signal output and photon numbers throughout the paper.

Supplementary Table 1

Sequence of the dsDNA samples

Sample 1:

20 bp separation (total length 60 bp)

5' - CCGTTGCGTT CGCTGGCGC/iCy3/ CCCGGCGCAT GTTACCGCGA CGGGCCAGGT ACACTAAGGG - 3'

5' - CCCTTAGTGT ACCTGGCCCG /iCy5/CGCGGTAAC ATGCGCCGGG AGCGCCAGCG AACGCAACGG - 3'

Sample 2:

30 bp separation (total length 90 bp)

5' - CCGTTGCGTT GGAGGGCTCC CGCTGGCGC/iCy3/ CCCGGCGCAT CCGAGTAGTC GTTACCGCGA CGGGCCAGGT GTTGAACGGG ACACTAAGGG - 3'

5' - CCCTTAGTGT CCCGTTCAAC ACCTGGCCCG /iCy5/CGCGGTAAC GACTACTCGG ATGCGCCGGG AGCGCCAGCG GGAGCCCTCC AACGCAACGG - 3'

Supplementary Note 1

Probability distribution of relative angles between emission dipoles

The "normal" distribution of errors on spherical coordinates is the solution to the diffusion equation on the surface of the sphere with initial condition that all probability is located in a single point. Like the normalized 2D Gaussian, this distribution on the sphere has three parameters: The two spherical coordinates of its center, plus its variance. We find this pdf here by solving the diffusion equation.

One may think of the diffusing entity as the orientation of a dipole. We are particularly interested in the error on the angle between two such dipoles, both of which come with errors on their orientation, i.e., both of which have a normal probability distribution on the sphere. With no loss of generality, we can find the probability distribution of angles between two such dipoles by keeping one of them fixed and letting the other one diffuse with correspondingly higher diffusion coefficient.

With no loss of generality, we can let the fixed dipole define the North Pole of the sphere. Then the angle between the two dipoles is the polar angle of the other, diffusing dipole, and its distribution at any time is found by solving the diffusion equation for the other dipole and integrating over the azimuth of the solution. With no loss of generality, we can also define this azimuth such that the initial orientation of the diffusing dipole has zero azimuth. Specifically, let the orientation of a dipole be given as a point Ω on the unit sphere.

$$\Omega = (\theta, \phi) = \begin{pmatrix} \cos\phi\sin\theta \\ \sin\phi\sin\theta \\ \cos\theta \end{pmatrix} \tag{1}$$

in spherical and Cartesian coordinates, respectively. The orientation of the fixed dipole defining the North Pole has coordinates $\Omega_1 = (\theta_1 = 0, \phi_1 = 0)$ at all times. The orientation of the other, diffusing dipole has coordinates $\Omega_0 = (\theta_0, \phi_0 = 0)$ at time $t = 0$.

Let $\rho(\Omega, t)$ denote the distribution of orientations Ω at time t that results from diffusion with coefficient D from initial orientation Ω_0. It satisfies the diffusion equation,

$$\frac{\partial}{\partial t}\rho(\Omega, t) = D\triangle\rho(\Omega, t) \ , \tag{2}$$

with initial condition $\rho(\Omega, 0) = \delta_{\Omega_0}(\Omega)$. This diffusion is restricted to the surface of the unit sphere, so the Laplace operator is, in spherical coordinates,

$$\triangle = \frac{1}{\sin\theta}\frac{\partial}{\partial\theta}\left(\sin\theta\frac{\partial}{\partial\theta}\right) + \frac{1}{\sin^2\theta}\frac{\partial^2}{\partial\phi^2} \ . \tag{3}$$

By separation of the variables, first of t and Ω, then of ϕ and θ, we find a

general solution in the form

$$\rho(\Omega, t) = \sum_{\ell=0}^{\infty} \sum_{m=-\ell}^{\ell} \rho_\ell^m e^{-\ell(\ell+1)Dt} Y_\ell^m(\Omega) \ . \tag{4}$$

For each value of ℓ and m, ρ_ℓ^m is a constant, while Y_ℓ^m is the spherical harmonic of degree ℓ and order m, explicitly,

$$Y_\ell^m(\Omega) = \sqrt{\frac{2\ell+1}{4\pi} \frac{(\ell-m)!}{(\ell+m)!}} P_\ell^m(\cos\theta) e^{im\phi} \ , \tag{5}$$

where P_ℓ^m is the associated Legendre Polynomial of degree ℓ and order m. With this definition of the spherical harmonic, it is orthonormal, i.e.

$$\int_{\theta=0}^{\pi} \int_{\phi=0}^{2\pi} Y_\ell^m \left(Y_{\ell'}^{m'}\right)^* d\Omega = \delta_{\ell\ell'} \delta_{mm'} \ . \tag{6}$$

In terms of the general solution, the initial value reads

$$\rho(\Omega, 0) = \sum_{\ell=0}^{\infty} \sum_{m=-\ell}^{\ell} \rho_\ell^m Y_\ell^m(\Omega) \ . \tag{7}$$

Multiplication by $(Y_\ell^m)^*$, integration over the surface of the unit sphere S_2, and use of the orthonormality of the spherical harmonics yields

$$\rho_\ell^m = (Y_\ell^m)^* (\Omega_0) \ . \tag{8}$$

Explicitly, the general solution becomes

$$\rho(\Omega, t) = \sum_{\ell=0}^{\infty} \sum_{m=-\ell}^{\ell} (Y_\ell^m)^* (\Omega_0) \, e^{-\ell(\ell+1)Dt} \, Y_\ell^m(\Omega) \ . \tag{9}$$

In terms of Legendre Polynomials, we may write it as

$$\rho(\Omega, t) = \sum_{\ell=0}^{\infty} \sum_{m=-\ell}^{\ell} \frac{2\ell+1}{4\pi} \frac{(\ell-m)!}{(\ell+m)!} P_\ell^m(\cos\theta_0) P_\ell^m(\cos\theta) \, e^{-\ell(\ell+1)Dt} \, e^{im(\phi-\phi_0)} \ . \tag{10}$$

We are particularly interested in the probability distribution of polar angles, since, as argued above, this is related to the probability distribution of relative orientations of dipoles, whose orientations are measured with normal distributed errors. This distribution emerges by integrating the previous expression over the azimuthal angle,

$$\begin{aligned} p_{\mathsf{pol}}(\theta) &= \sin\theta \int_0^{2\pi} d\phi \, \rho(\Omega, t) \\ &= \sin\theta \sum_{\ell=0}^{\infty} (\ell + \tfrac{1}{2}) P_\ell^0(\cos\theta_0) P_\ell^0(\cos\theta) e^{-\ell(\ell+1)Dt} \ , \end{aligned} \tag{11}$$

and using the fact that the emission dipole moments are not directional, such that the distribution of relative angles $\psi \in [0, \pi/2]$ becomes

$$p_{\text{rel}}(\psi) \quad = \quad p_{\text{pol}}(\psi) + p_{\text{pol}}(\pi - \psi) \ . \tag{12}$$

We compare this distribution to the measured relative orientations from emission dipoles (Fig. 3i,j). However, due to the relatively large angular separation between the probes compared to the noise in our measurement, this function is practically indistinguishable from a normal distribution throughout this paper.

Supplementary References

1. Mortensen, K. I., Churchman, L. S., Spudich, J. A. & Flyvbjerg, H. Optimized localization analysis for single-molecule tracking and super-resolution microscopy. *Nat. Methods* **7,** 4–10 (2010).

2. Dolghih, E. Theoretical studies of dye-labeled DNA systems with applications to fluorescence resonance energy transfer. (University of Florida, 2009).

3. Pertsinidis, A., Zhang, Y. & Chu, S. Subnanometre single-molecule localization, registration and distance measurements. *Nature* **466,** 647–651 (2010).
