# Supplementary material for: Optimized measurements of separations and angles between intra-molecular fluorescent markers
Source: Nat Commun. 2015 Oct 16;0:8621. doi: 10.1038/ncomms9621 (PMC4634324; doi:10.1038/ncomms9621)
Supplement: Supplementary Software — Python (python.org) code for the maximum likelihood estimation of position and orientation of fixed dipole emitters positioned close to the coverslip and imaged close to the design focal plane onto an EMCCD camera in a TIRF microscopy experiment (Methods). To this end, the theoretical point spread function is fitted to experimental intensity distributions. To speed up fitting, an accurate analytical approximation to the theoretical point spread function is used (Methods, Supplementary Figs. 10 and 11). The maximum likelihood estimates of the parameters are returned along with their theoretical uncertainties. For details, see the readme file enclosed with the Supplementary Software. [file ncomms9621-s2.zip › SupplementarySoftware/readme.pdf]

Supplementary Software: Readme

for

"Optimized measurements of separations and angles between intra-molecular fluorescent markers"

by

Kim I. Mortensen, Jongmin Sung,
Henrik Flyvbjerg, and James A. Spudich

September 8, 2015

Python script performing the analysis of diPOLE, i.e. maximum likelihood estimation of location and orientation of a fixed dipole along with the objective's distance from the design focal plane.

The file *diPOLE.py* contains the Python (python.org) code for the calculation of the PSF for fixed dipole emitters imaged close to the design focal plane. The class *dipdistr* contains a function that calculates the exact PSF for given orientation of the dipole. It calls the module *numint.py* that contains some functions for numerical integration. By initiation of the class, an automatic check for already saved normalization parameters is done — if not found, normalization is performed and the parameters are saved to the file *dipolenorm.dat* in the working directory. Any working directory should be used to analyze data from a single wavelength only. Also at initiation, the integrals of the cumulant approximation of the theoretical PSF are calculated for each value of the distance to the design focal plane. Code for the maximum likelihood estimation based on this theoretical PSF is given in the class *MLEwT*. It uses the class *LogLikelihood* to perform the maximum likelihood estimation of the position coordinates, the orientation of the probe, the number of background photons per pixel, the total photon number, and the distance to the design focal plane (see end of code for an example that reads simulated data from the file *data.txt* and estimates the position and orientation of a fixed fluorophore). By using the class *MLEwTcovar*, the code also returns the uncertainties for the estimated parameters.
